# Single-nuclei transcriptome analysis of the shoot apex vascular system differentiation in *Populus*

**DOI:** 10.1242/dev.200632

**Published:** 2022-10-17

**Authors:** Daniel Conde, Paolo M. Triozzi, Wendell J. Pereira, Henry W. Schmidt, Kelly M. Balmant, Sara A. Knaack, Arturo Redondo-López, Sushmita Roy, Christopher Dervinis, Matias Kirst

**Affiliations:** ^1^ School of Forest, Fisheries and Geomatics Sciences, University of Florida, Gainesville, FL 32611, USA; ^2^ Centro de Biotecnología y Genómica de Plantas, Universidad Politécnica de Madrid – Instituto Nacional de Investigación y Tecnología Agraria y Alimentaria (INIA-CSIC), Madrid 28223, Spain; ^3^ Wisconsin Institute for Discovery, University of Wisconsin, Madison, WI 53715, USA; ^4^ Department of Computer Sciences, University of Wisconsin, Madison, WI 53792, USA; ^5^ Department of Biostatistics and Medical Informatics, University of Wisconsin, Madison, WI 53792, USA; ^6^ Genetics Institute, University of Florida, Gainesville, FL 32611, USA

**Keywords:** *Populus*, Vascular development, scRNA-seq, Developmental trajectory, Shoot apex, Cell differentiation

## Abstract

Differentiation of stem cells in the plant apex gives rise to aerial tissues and organs. Presently, we lack a lineage map of the shoot apex cells in woody perennials – a crucial gap considering their role in determining primary and secondary growth. Here, we used single-nuclei RNA-sequencing to determine cell type-specific transcriptomes of the *Populus* vegetative shoot apex. We identified highly heterogeneous cell populations clustered into seven broad groups represented by 18 transcriptionally distinct cell clusters. Next, we established the developmental trajectories of the epidermis, leaf mesophyll and vascular tissue. Motivated by the high similarities between *Populus* and *Arabidopsis* cell population in the vegetative apex, we applied a pipeline for interspecific single-cell gene expression data integration. We contrasted the developmental trajectories of primary phloem and xylem formation in both species, establishing the first comparison of vascular development between a model annual herbaceous and a woody perennial plant species. Our results offer a valuable resource for investigating the principles underlying cell division and differentiation conserved between herbaceous and perennial species while also allowing us to examine species-specific differences at single-cell resolution.

## Teaser

**Summary:** Single-nuclei analysis identified regulatory genes of cell differentiation in poplar apex, establishing a comparison between a model annual herbaceous and a woody perennial plant species.

## INTRODUCTION

In multicellular organisms, body parts differentiate from pluripotent stem cells. In plants, these cells are present in meristematic tissues, including the shoot apical meristem (SAM). Except for the hypocotyl and cotyledons, the SAM is responsible for generating all aerial parts of the plant. To fulfill this function, the meristem must balance the self-renewal of a reservoir of stem cells and organ initiation. Most of the knowledge about the signaling networks that regulate this balance comes from the model species *Arabidopsis* [*Arabidopsis thaliana* (L.) Heynh.]. The *Arabidopsis* SAM is divided into three cell layers. The L1 layer derivatives give rise to the epidermis of shoots, leaves and flowers, the L2 layer provides the mesophyll tissue and germ cells and the L3 layer contributes to the vascular tissues and pith (Zhang et al., 2021). The *Arabidopsis* SAM can also be divided into distinct cell domains or zones. Stem cells reside in the central zone (CZ). The peripheral zone (PZ), in which cells divide more frequently than in the CZ, is responsible for organ initiation. The rib meristem (RM) gives rise to central tissues of the shoot axis (Dinneny and Benfey, 2008; Holt et al., 2014).

The stem cells of the vegetative shoot apex undergo divisions to generate transitioning cells that eventually differentiate into lateral organs such as leaves and vasculature. A fast vegetative growth characterizes annual plants like *Arabidopsis*. After perceiving specific environmental signals, *Arabidopsis* SAM initiates the transition from vegetative to reproductive phase, ceasing growth and developing flowers (Kinoshita et al., 2020). Meristem activity of perennial differs from that of annuals in several aspects, including an extended juvenile period and seasonally synchronized vegetative growth-dormancy cycles (Singh et al., 2021). Although well explored in *Arabidopsis*, we lack an understanding of cell lineage origin and developmental trajectory that give rise to shoot apex cells in woody perennial species such as *Populus*. Previous profiling studies in *Arabidopsis* used reporter genes to purify domain-specific cell populations (Tian et al., 2019; Yadav et al., 2014), an approach that underestimates cell complexity as it restricts the analysis to the expression domains of these markers. The development of microfluidic-based single-cell RNA-sequencing (scRNA-seq) methods established the foundation for quantifying the complete transcriptome at cellular resolution and enabled the characterization of novel cell subpopulations in heterogeneous tissues in plants (Birnbaum, 2018; Mironova and Xu, 2019; Rich-Griffin et al., 2020). Moreover, scRNA-seq analysis allows ordering individual cells in an inferred trajectory to reveal the lineages that determine plant form. This approach permitted, in *Arabidopsis*, the discovery of the developmental trajectories from SAM proliferating cells to the formation of new organs (Zhang et al., 2021). This approach has also been used in *Arabidopsis* to explore the root development (Denyer et al., 2019; Ryu et al., 2019; Shulse et al., 2019; Zhang et al., 2019). However, methods of protoplast isolation used in *Arabidopsis* are not immediately applicable to many tissues and species. Consequently, a high-resolution and cell type-specific gene expression map of the shoot apex of woody perennials is still lacking, resulting in a limited understanding of the regulatory mechanism involved in the organ differentiation from SAM stem cells in these species.

Here, we applied a novel approach to isolate nuclei from complex plant tissues (Conde et al., 2021) to dissect the transcriptome profile of the hybrid poplar (*Populus tremula*×*alba*) vegetative shoot apex at single-cell resolution. We inferred the developmental trajectories that occur during the generation of new tissues in the SAM. We then assessed the development of the primary vasculature, a process largely unexplored in woody perennial species, and identified regulators of the vascular tissue differentiation. Finally, we applied a pipeline for interspecies comparison of single-cell transcriptome data. We contrasted the developmental trajectories of primary phloem and xylem formation in *Arabidopsis* and *Populus* with this pipeline, establishing the first comparison between primary vasculature development at the single-cell level between a model annual herbaceous and a woody perennial plant species.

## RESULTS

### An atlas of *Populus* vegetative shoot apex cells

**Fig. 1.**
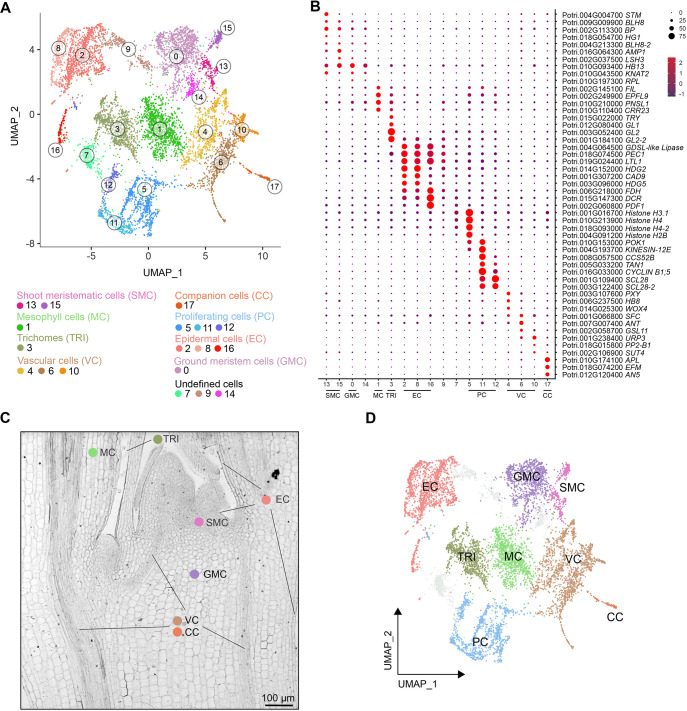
**Characterization of cell populations in the *Populus* vegetative shoot apex.** (A) Visualization of the 18 cell clusters using UMAP. Dots, individual cells; *n*=8324 cells; color, cell clusters. (B) Expression pattern of previously characterized cell type marker genes in the *Populus* apex cell clusters. Dot diameter is the proportion of cluster cells expressing a given gene. The complete list of genes used for cluster annotation is given in Table S2. (C) A longitudinal section of a *Populus* apex shows the spatial location of the population of annotated cells. (D) Visualization of the eight cell clusters using UMAP. Colors represent the cell types. Scale bar: 100 μm.

We harvested 20 shoot apices from 3-week-old *in vitro* grown hybrid poplar (*Populus tremula*×*alba*) plants and performed high-throughput, microfluidic-based single-nuclei RNA-sequencing (snRNA-seq) using the 10x Genomics Chromium technology as we described previously (Conde et al., 2021). We used Asc-Seurat v.2.1 (Pereira et al., 2021), a comprehensive web application that encapsulates a series of tools for scRNA-seq data analysis. We removed potentially empty (containing only ambient RNA and not nuclei) gel bead-in-emulsions (GEMs) by requiring a minimum of 1000 expressed genes detected per nucleus and excluded those nuclei with more than 7000 because those are potentially multiplets (Table S1; Fig. S1). We captured 8324 high-quality nuclei and obtained on average 59,475 reads per nucleus. An average of 3618 unique molecular identifiers (UMIs), corresponding to the expression of 2477 genes, were detected per nucleus. Overall, 31,214 genes were detected (Table S1). After processing the data and applying a graph-based clustering approach from Seurat (Butler et al., 2018), 18 distinct clusters were uncovered (Fig. 1A; Fig. S1). To annotate each cluster, we assessed the abundance of transcripts of *Populus* homologous to well-known cell type marker genes in *Arabidopsis* for the different domains of the vegetative or reproductive shoot apex (Tian et al., 2019; Yadav et al., 2014; Zhang et al., 2021) (Table S2). We also evaluated the expression of homologous genes that, in *Arabidopsis*, are mainly expressed in different cell types of the vegetative and the reproductive shoot apex (Tian et al., 2019; Yadav et al., 2014). Moreover, we identified cell markers for each cluster detected in the *Populus* shoot apex (Table S3) and explored those for which biological functions or expression patterns have previously been well characterized. Based on these three sources of information, we selected the *Populus* tissue-specific markers used to annotate the clusters (Table S2). Clustering annotation revealed populations of eight cell types: trichomes (TRI), mesophyll cells (MC), epidermal cells (EC), shoot meristematic cells (SMC), proliferating cells (PC), vascular cells (VC), companion cells (CC) and ground meristem cells (GMC) (Fig. 1A-D).

The MC population consisted of one cluster (cluster 1) (Fig. 1A), in which *Populus* homologs to *Arabidopsis* markers for the mesophyll, such as *EPIDERMAL PATTERNING FACTOR LIKE-9* (*EPFL9*) (Kondo et al., 2010; Lopez-Anido et al., 2021), *CHLORORESPIRATORY REDUCTION 23* (*CRR23*) or *PHOTOSYNTHETIC NDH SUBCOMPLEX L1* (*PNSL1*) (Zhang et al., 2021) were predominantly expressed (Fig. 1B; Table S2). Photosynthesis and small molecule metabolic processes are the most enriched gene ontologies using the *de novo* identified marker genes of cluster 1 (Tables S3, S4). The epidermal-specific genes *Li-TOLERANT LIPASE 1* (*LTL1*) (Yadav et al., 2014), *HOMEODOMAIN GLABROUS 2* (*HDG2*) (Yadav et al., 2014) and *PROTODERMAL FACTOR 1* (PDF1) (Abe et al., 1999) were detected in the EC population (i.e. L1 layer), which consisted of clusters 2, 8 and 16 (Fig. 1B). The SMC population consisted of two clusters, 13 and 15 (Fig. 1B). Transcripts for *Populus* homologs to the *Arabidopsis SHOOT MERISTEMLESS* (*STM*) and *BREVIPEDICELLUS* (*BP*), which are required for the establishment and maintenance of the *Arabidopsis* SAM (Clark et al., 1996; Gallois et al., 2002; Lincoln et al., 1994; Long et al., 1996), and other related homeodomain genes, *HOMEOBOX GENE 1* (*HG1*; also known as ATH1) (Rutjens et al., 2009) and *BEL1-LIKE HOMEODOMAIN 8* (*BLH8*) (Ung and Smith, 2011), were highly enriched in this cell population (Fig. 1B). We annotated three clusters (5, 11, 12) as the PC population because transcripts for homologs to cell cycle-related genes such as *HISTONE H4* (*H4*), *CELL CYCLE SWITCH PROTEIN 52 B* (*CCS52B*), *CYCLIN B1;5* (*CYCB1;5*) or *SCARECROW-LIKE 28* (*SCL28*) (Goldy et al., 2021; Menges et al., 2002) were overrepresented (Fig. 1B). The VC population was composed of four clusters (4, 6, 10 and 17) (Fig. 1A), in which genes involved in xylem and phloem differentiation were expressed. For example, transcripts of the *Populus* homolog to the xylem gene *PHLOEM INTERCALATED WITH XYLEM* (PXY) (Etchells and Turner, 2010; Fisher and Turner, 2007; Shi et al., 2021) and the phloem gene *PHLOEM PROTEIN 2-B1* (*PP2-B1*) (Dinant et al., 2003) were markedly overrepresented in clusters 4 and 10, respectively (Fig. 1B). Homologs to markers for cambium stem cells, such as *AINTEGUMENTA* (*ANT*) (Randall et al., 2015), were enriched in cluster 6. The CC population (cluster 17) was observed in a highly distinct cluster on the UMAP plot, agreeing with their specific physiological functionalities and unique expression profiles (Fig. 1A). Transcripts for *Populus* homologs to CC marker genes such as *ARATHNICTABA 5* (*AN5*) and *EARLY FLOWERING MYB* (*EFM*) (Dinant et al., 2003; Yan et al., 2014; Zhang et al., 2021) are highly accumulated in cluster 17 (Fig. 1B). In *Arabidopsis*, the use of protoplasts in the single-cell analysis of developing leaves failed to identify the cluster corresponding with TRI, as these cells are not isolated in the process (Tenorio Berrío et al., 2022; Zhang et al., 2021). The expression of well characterized regulators of TRI formation, such as *GLABROUS/GLABRA1* (*GL1*), *GL2*, and *TRIPTYCHON* (*TRY*) (Hülskamp, 2004) was restricted to cluster 3 and, accordingly, we annotated it as TRI (Fig. 1A-D). Finally, we also assessed the functional annotation of *de novo* identified marker genes identified for the cluster 0 (Table S3), which contains a large number of cells. More specifically, cells of this cluster contain transcriptome signatures of stem cells, such as an enrichment in the expression of *KNOTTED-LIKE FROM ARABIDOPSIS THALIANA 2* (*KNAT2*) or *LIGHT SENSITIVE HYPOCOTYLS 3* (*LSH3*) (Fig. 1B) (Scofield et al., 2014; Zhang et al., 2021). Moreover, the expression of *RPL* is restricted to a group of cells located in cluster 0 (Fig. 1B). This gene regulates rib zone cell division and growth (Bencivenga et al., 2016). *HB13* is also a marker of cluster 0. In *Populus* stem, it has been described as expressed in undifferentiated parenchyma tissue (Hanson et al., 2002). Based on this, we annotated cluster 0 as GMC – i.e. the tissue located below the stem cells in the apex, which also includes the RM. These cells are highly represented in the apex. Also, the vascular cambium arises when the cells of the interfascicular parenchyma of the ground tissue located between the vascular bundles of the apex dedifferentiate and divide, connecting the procambium and completing the radial arrangement of the vascular cambium. The above results indicate that the vegetative shoot apex is composed of highly heterogeneous cells and suggests that many regulatory mechanisms of the vegetative SAM and shoot apex are conserved in *Populus* and *Arabidopsis*.

### Developmental trajectories of the epidermis, mesophyll and vascular tissue

**Fig. 2.**
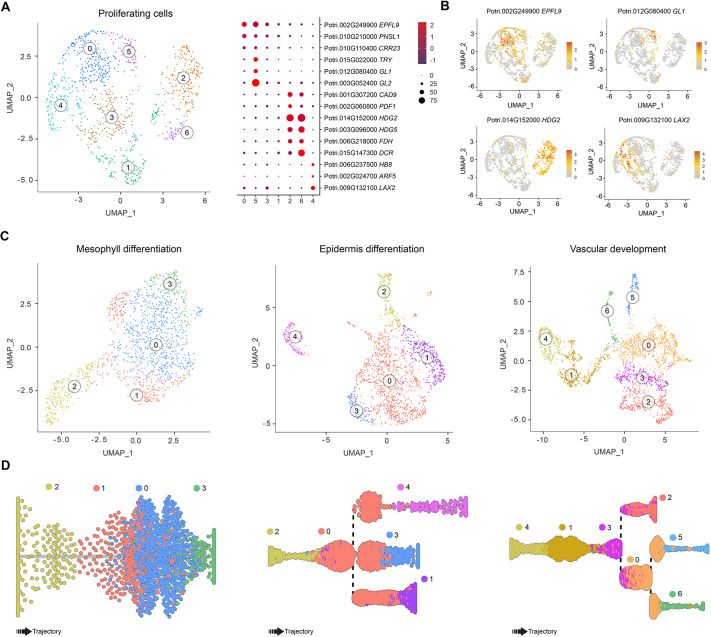
**Characterization of the proliferating cell population and developmental trajectories for mesophyll cells, epidermis cells and vascular cells.** (A) Sub-clustering clusters 5, 11 and 12 reveal cell type-specific proliferating cell (PC) populations. (B) The expression pattern of well-characterized marker genes showed the trichomes and mesophyll (MC)-specific (sub-clusters 5 and 0), vascular (VC)-specific (sub-cluster 4) and epidermis (EC)-specific (sub-clusters 2 and 6) cell clusters within the PC population. (C) Re-clustering of MC, EC and VC clusters with their corresponding sub-clusters of PC population. (D) Slingshot analysis showing the position of the sub-clusters during the epidermis, mesophyll and vascular tissue differentiation trajectory or pseudotime.

Single-cell RNA-sequencing and analysis have recently allowed the identification of the developmental trajectories of the epidermis, mesophyll and vasculature differentiation in the *Arabidopsis* vegetative shoot apex (Zhang et al., 2021). As previously described for the *Arabidopsis* shoot apex, we identified a large population of PC in *Populus*. Following the same approach performed by Zhang et al. (2021), we dissected the PC population to identify the specific sub-groups of EC (L1 layer), MC (L2 layer) and VC (L3 layer) proliferating cells. Cells belonging to clusters 5, 11 and 12 (Fig. 1A) were re-clustered at a higher resolution to perform this dissection, revealing seven sub-clusters (Fig. 2A). Sub-clusters 0 and 5 exhibited high transcript levels of *EPFL9* (a marker of MC) and *GL1* (a marker of TRI), respectively (Fig. 2B). Sub-clusters 2 and 6 showed high expression levels of the epidermal cell markers *PDF1* and *FIDDLEHEAD* (*FDH*), respectively (Abe et al., 1999; Yephremov et al., 1999; Zhang et al., 2021) (Fig. 2B). In contrast, the vascular meristem markers *LIKE AUXIN RESISTANT 2* (*LAX2*) (Péret et al., 2012) and *HOMEOBOX GENE 8* (*HB8*) (Baima et al., 1995; Jouannet et al., 2015) were highly expressed in sub-cluster 4 (Fig. 2B). The transcript profile of these marker genes indicates that the PC population contains the cells that differentiate into the epidermis, mesophyll and VC. Next, we delineated the developmental trajectories that give rise to these cells. In Asc-Seurat, we re-grouped the EC (clusters 2, 8 and 16), MC (cluster 1) and VC clusters (clusters 4, 6, 10 and 16) separately, with the PC clusters (clusters 5, 11, 12) (Fig. 1A). Then we excluded the PC sub-clusters that did not belong to the EC, MC and VC, based on the expression of markers for each cell type (Fig. 2A), to generate the final clustering of EC, MC and VC with their corresponding PC sub-clusters (Fig. 2C). Then we applied Slingshot v.1.8 (Street et al., 2018) to infer the developmental trajectory for the epidermis, mesophyll and vascular tissue differentiation (Fig. 2D). Identifying the proliferating cells with EC, MC and VC transcriptional signatures paved the way to investigate how SAM stem cells differentiate into the distinct new tissues formed in the *Populus* shoot apex.

### Integration and annotation of *Populus* and *Arabidopsis* vasculature single-cell data

The similarity between the cell type population identified in *Populus* and *Arabidopsis* shoot apex motivated the exploration of the conservation and divergence of the molecular mechanisms of cellular differentiation between these two species, focusing on the primary vascular development. For this comparison, we used single-cell gene expression data from shoot apex vasculature of *Arabidopsis* (Zhang et al., 2021). Clustering of VC from both species showed a similar cell population structure (Fig. S2A), according to the expression patterns of previously well-characterized markers for PC, phloem, xylem and CC (Fig. S2B). Integrating both datasets using Seurat's integration method (Butler et al., 2018) requires a one-to-one homolog gene relationship between *Populus* and *Arabidopsis*. The complex history of whole-genome duplications, chromosomal rearrangements, and tandem duplications in *Populus* resulted in many duplicated genes, which implies that there are several *Populus* homologs for each *Arabidopsis* gene (Tuskan et al., 2006). To create a one-to-one homolog genes list required for the integration, we applied a phylogenomic approach to defining a high-confidence gene list of 9842 *Arabidopsis* and *Populus* pairs of homologs (Table S5). The expression of these genes was used to integrate the single-cell expression data of *Populus* and *Arabidopsis* shoot apex vasculature. Homology between the remaining genes of *Populus* (not present in the one-to-one homolog genes list) and *Arabidopsis* genes was established based on the most recently inferred relationships, available in Phytozome (Goodstein et al., 2012) (Table S6). After the data integration, we used this complete list to explore conserved and divergent pathways. Table S6 contains 23,732 *Populus* genes with the corresponding homolog in *Arabidopsis*. After the integration, the pipeline used the gene IDs in the column ‘*Arabidopsis*’ to explore the expression of *Arabidopsis* and *Populus* genes. To differentiate the expression of *Populus* paralogs that share a common *Arabidopsis* homolog, a dot followed by a number was added to the *Arabidopsis* ID of the corresponding *Arabidopsis* homolog (Table S6). For this reason, in the supplementary tables generated after the integration (Table S7 to Table S15), in the ‘*Populus*’ column, an *Arabidopsis* ID, followed by a dot and a number can be seen when the *Populus* gene has a homolog in *Arabidopsis*. The actual *Populus* ID can be inferred by searching that gene ID in Table S6. Only when the *Populus* gene does not have an *Arabidopsis* homolog associated in Table S6 does the *Populus* ID appear in the column ‘*Populus’*.

**Fig. 3.**
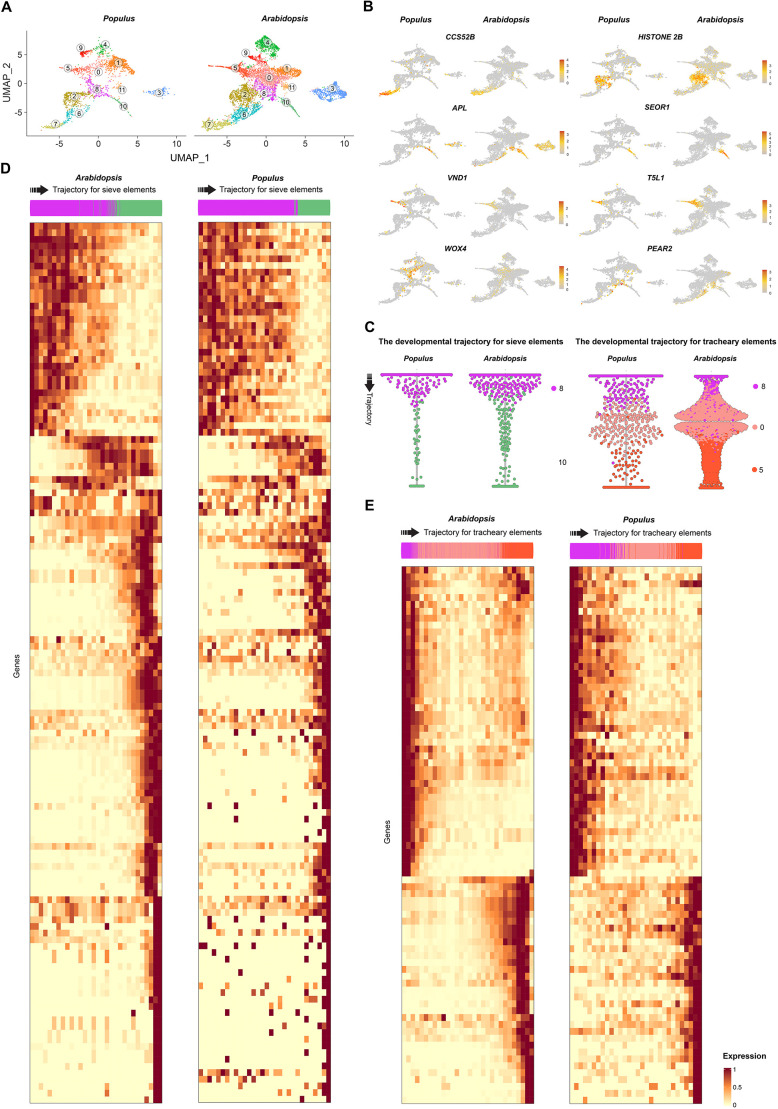
**Characterization of the developmental trajectory of the primary vasculature development.** (A) Clustering of the primary vasculature after integrating *Arabidopsis* and *Populus* snRNA-seq data from the apex. (B) The expression of well-known markers identified the proliferating cells (CCS52B, HISTONE 2B), primary phloem (APL, SEOR1) and tracheary elements (VND1, T5L1). (C) Slingshot was used to run the trajectory for the primary phloem and tracheary elements in the *Populus*-*Arabidopsis* integrated data. Trade-seq was used to identify genes involved in the cell type specification. (D) Heatmap with the expression of genes associated with the developmental trajectories of sieve elements in both *Populus* and *Arabidopsis*, which shared the same expression pattern based on their positive correlated expression between species, calculated by dividing the trajectory into 30 bins. (E) Heatmap with the expression of the genes associated with the developmental trajectories of tracheary elements in both *Populus* and *Arabidopsis* that shared the same expression pattern based on their positive correlated expression between species, calculated by dividing the trajectory into 30 bins. See Fig. S6 for more details.

The UMAP visualization of the final integrated data showed that most cells were distributed in common cell type clusters between both species (Fig. 3A; Fig. S3). Three clusters specific to *Arabidopsis* were excluded during the integration step, two of which were enriched for stress-responding genes, and a small cluster (Fig. S3A,B). It is not possible to infer whether the clusters containing stress-responsive genes are only present in *Arabidopsis* owing to differences between the species, or whether they are the result of the induction of expression of stress-responsive genes due to the protoplasting process. Overall, VC were distributed in 12 clusters after the integration. We identified 3 clusters (2, 6 and 7) containing the PC, based on the expression of cell cycle-related genes such as *CCS52B* or *HISTONE 2B*. Based on the expression pattern of markers used in *Arabidopsis* (Zhang et al., 2021) for the sieve elements [*ALTERED PHLOEM DEVELOPMENT* (*APL*) and *SIEVE ELEMENT OCCLUSION-RELATED 1* (*SEOR1*)] and tracheary elements [*VASCULAR RELATED NAC-DOMAIN PROTEIN 1* (*VND1*) and *TARGET OF MONOTEROS 5-LIKE 1* (*T5L1*)], we annotated clusters 10 and 5 as sieve and tracheary elements cells, respectively (Fig. 3B). Interestingly, the expression profiles of *PHLOEM EARLY DOF 2* (*PEAR2*) and *WUSCHEL RELATED HOMEOBOX 4* (*WOX4*) suggest induction of these genes before sieve and tracheary elements differentiation, respectively. Next, we used Slingshot to establish the overall developmental trajectory of the integrated vasculature data (Fig. S3C) and determine the clusters associated with cell lineages that result in the formation of sieve and tracheary elements. The overall developmental trajectory pointed to clusters 8 and 10 as being involved in sieve element trajectory, whereas 8, 0 and 5 participate in tracheary element differentiation (Fig. S3).

### Comparison between *Populus* and *Arabidopsis* primary vasculature development at single-cell resolution

To uncover conserved and divergent regulatory mechanisms involved in the sieve and tracheary element differentiation between *Populus* and *Arabidopsis*, we inferred their developmental trajectories after the data integration. We used clusters 8 and 10 for the sieve element trajectory and 8, 0 and 5 for the tracheary element differentiation (Fig. 3C). Then, we applied a generalized additive model regression implemented in tradeSeq (Van den Berge et al., 2020) to identify genes for which expression changes significantly along the trajectories. These genes are putative regulators of the cell differentiation process occurring during the formation of the sieve and tracheary elements, or genes controlled by these regulators. We identified 576 differentially expressed genes (DEGs) in the phloem and 191 DEGs in the xylem of the *Populus* developmental trajectory (FDR≤0.01). For phloem, out of 576 DEGs, we identified confidently (based on Table S6) the *Arabidopsis* homolog for 431. Of note, 60% of these (259 genes) were DEGs in the *Arabidopsis* trajectory (Table S7; Fig. S4). For xylem, 81% (129 genes) were also DEGs in *Arabidopsis* (Table S8; Fig. S5). To identify which genes present the same expression pattern in both species during cell differentiation, we divided each phloem and tracheary element trajectory into 30 bins with an equal number of cells. Bins were constructed continuously along the trajectory such that the first bin contained the first one-thirtieth cells, etc., until the 30th bin. Binning the cell data allowed us to quantify gene expression in regular segments along the trajectory in each species and, posteriorly, to calculate the gene expression correlation between *Populus* and *Arabidopsis* along the trajectories. Out of the 259 common DEGs for phloem development, 51% (132 genes) were significantly correlated (Corr.≥0.5; FDR≤0.01) between species (Table S9; Fig. 3D). Among 129 common DEGs for the xylem, 60% (78 genes) were correlated (Table S10; Fig. 3E). Correlated gene IDs and their expression along the trajectories are shown in Fig. S6. This comparative single-cell analysis highlighted the conservation of key regulatory genes that control sieve element differentiation, previously identified in *Arabidopsis*, including *CLV3/EMBRYO SURROUNDING REGION 45* (*CLE45*) (Depuydt et al., 2013; Shimizu et al., 2015), *APL* (Bonke et al., 2003), *SEOR1*, *HIGH CAMBIAL ACTIVITY2* (*HCA2*) (Guo et al., 2009; Kondo et al., 2016) or *LATERAL ROOT DEVELOPMENT 3* (*LRD3*) (Ingram et al., 2011) (Fig. S6; Table S9). *APL* has been identified as a marker of sieve elements and CC in *Populus* stem, indicating its conserved function (Chen et al., 2021). Among highly correlated but less characterized conserved genes implicated in phloem differentiation, we detected *HOMEOBOX PROTEIN 33* (*HB33*), *NAC DOMAIN CONTAINING PROTEIN* 57 and 75 (*NAC057* and *NAC075*), or *LONESOME HIGHWAY LIKE 1* (*LHL1*).

Correlated genes for tracheary element differentiation included well-known regulators of xylem development such as *MONOPTEROS* (*MP*), *VND1*, *ACAULIS 5* (*ACL5*), *PXY* or *T5L1*, although some of these regulators (e.g. *MP* and *PXY*) are not specific for tracheary elements during xylem formation (Etchells and Turner, 2010; Fisher and Turner, 2007; Smetana et al., 2019).

The comparison between the *Arabidopsis* and *Populus* trajectories also identified genes for which the gene expression changes significantly in phloem and xylem only in *Populus* (Tables S11 and S12; Figs S7 and S8)*.* We identified transcription factors related to phytohormones implicated in vascular formation, including auxin (*AUXIN RESPONSE FACTOR 2*) and cytokinin signaling (*CYTOKININ-RESPONSIVE GROWTH REGULATOR*). Homeodomain transcription factors (*HOMEOBOX PROTEIN 16*) or NAC domain-containing transcription factors (*NAC089*) were also found only in the *Populus* trajectories.

Our results suggest that the transcriptional programs of the primary vasculature formation identified in the model species *Arabidopsis* are conserved in perennial woody plants. However, a subset of genes significantly related to vasculature differentiation appears to be unique to *Populus* in their association to this developmental program. This observation highlights the relevance of our datasets to identifying previously unexplored cell differentiation mechanisms in both model species. This pipeline can also be applied to compare other developmental programs occurring in the shoot apex, such as leaf and epidermis differentiation.

### Comparison between *Populus* and *Arabidopsis* procambium

**Fig. 4.**
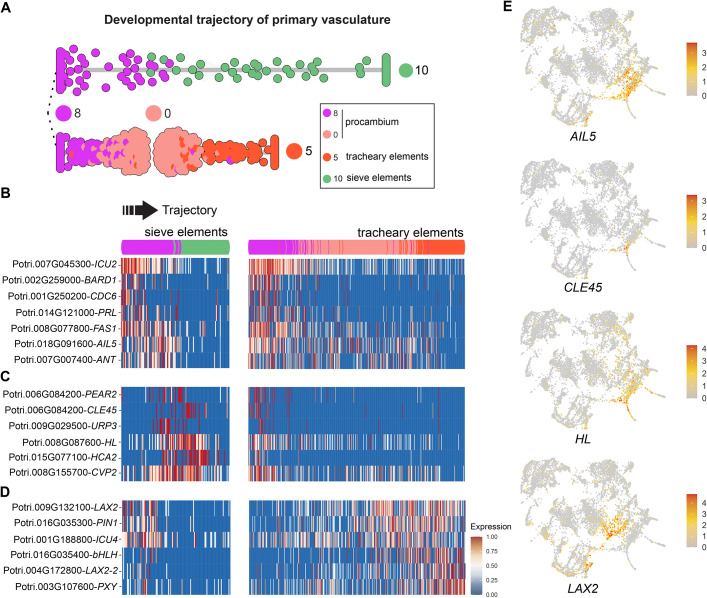
**Identification of procambium cell regulatory genes.** (A) Developmental trajectory of phloem (cluster 10) and tracheary elements (cluster 5) from the procambial cells (cluster 0 and 8) inferred by Slingshot. (B,C) A subset of the genes induced in cluster 8 of *Populus*, containing marker genes of vascular meristem, such as *ANT* (B), and phloem cell initiation, such as *PEAR2* (C). (D) A subset of genes induced in cluster 0 of *Populus*, containing genes involved in auxin signaling such as *LAX2* and *PIN1*. (E) These putative regulators show a very specific expression in the vasculature in the *Populus* apex. Cluster numbers and colors are as in Fig. 3A.

The procambium remains one of the most understudied plant tissues due to the challenge of dissecting the cell population derived from the SAM but not yet differentiated into phloem and xylem. In the integrated data of the VC, we identified three clusters corresponding to the PC (Fig. 3A,B). Dividing cells have a specific transcriptome signature and they are therefore clustered together during the data analysis. In addition to containing the proliferating cells of the procambium that are undergoing differentiation to phloem and xylem, these clusters may contain other dividing cells of the vasculature such as xylem or phloem cells undergoing further stages of differentiation. Hence, we cannot determine that PC of vasculature are procambial cells exclusively. However, as suggested by the trajectory of the VC (Fig. S3C) and the trajectories detected in *Arabidopsis* (Zhang et al., 2021), clusters 0 and 8 are transcriptionally similar to PC, and they contain the precursors for the phloem and xylem formation. However, these cells were not dividing at the time of sampling, based on the expression of cell-cycle marker genes (Fig. 3B). These two clusters are therefore suitable to study the vegetative shoot apex procambial cell biology (Fig. 4A).

To select genes potentially required for procambial cell maintenance, we identified DEGs in clusters 0 and 8 in *Populus* by comparing their transcript abundance to the other ten cell clusters generated after the data integration (Fig. 3A; Fig. S3). We detected 417 DEGs, primarily expressed in these two clusters (FDR≤0.05) (37 for cluster 0, 375 for cluster 8, and 5 in both Tables S13 and S14). For these 417 *Populus* genes, we identified 361 *Arabidopsis* homologs, of which 164 were also significantly induced in the *Arabidopsis* procambium (clusters 0 and 8 of the integrated data, Table S15).

We can distinguish two distinct expression patterns among the genes significantly induced in cluster 8. On one side, we have the genes for which expression is confined to cluster 8, but reduced in the transition to other clusters in the trajectory (Fig. 4B), suggesting that these genes are involved in maintaining the identity of these cells. Among these genes, we found *ANT*, *AINTEGUMENTA-like 5* (*AIL5*) and *INCURVATA 2* (*ICU2*) (Fig. 4B, Table S15). *ANT* is mainly expressed in the vascular cambium in *Arabidopsis* and *Populus* stem (Dewitte et al., 2007; Etchells et al., 2015; Randall et al., 2015; Schrader et al., 2004). The second pattern corresponds to genes expressed in the transition from cluster 8 to cluster 10 – the transition from procambium to phloem cells (Fig. 4C). Genes in that group included *HCA2*, *COTYLEDON VASCULAR PATTERN 2* (*CVP2*), which acts early in vasculature patterning during embryogenesis in *Arabidopsis* (Carland and Nelson, 2004), *PEAR2*, *CLE45* or *UAS-TAGGED ROOT PATTERNING3* (*URP3*). This evidence supports that cluster 8 contains procambial and phloem precursor cells (Miyashima et al., 2019; Waki et al., 2013). Some of these gene expression patterns are shared with *Arabidopsis* (Table S15).

In agreement with the premise that cluster 0 contains procambial and xylem cell precursors in *Populus*, we identified *WOX4* and *PXY* expression significantly induced in this cluster. In the *Arabidopsis* root, auxin signaling is required for the xylem differentiation (Smetana et al., 2019). We found that *Populus* homologs to *PIN1* and *LAX2* are induced in cluster 0 and that their expression is expanded to the xylem cells (Fig. 4D), suggesting that these genes are involved in xylem differentiation. Remarkably, the expression of *MP*, expressed in vascular cambium in the root (Smetana et al., 2019), occurs later in the xylem development in the shoot apex of *Populus* and *Arabidopsis* (Fig. S6). This observation suggests a more specific role of *MP* in xylem differentiation than procambium formation in the shoot apex primary vasculature. We also found a *Populus* homolog to *PHABULOSA* (*PHB*) induced in cluster 0, with a similar expression pattern to *PIN1*. *INCURVATA 4* (*ICU4*) promotes vascular development in *Arabidopsis* (Ochando et al., 2008), and has been related to xylem formation during secondary growth in *Populus* (Du et al., 2011) and xylem differentiation in *Zinnia elegans* (Ohashi-Ito and Fukuda, 2003) (Fig. 4B; Table S14). This gene is also induced in cluster 0, indicating that it contains procambial cells and precursors of xylem development.

**Fig. 5.**
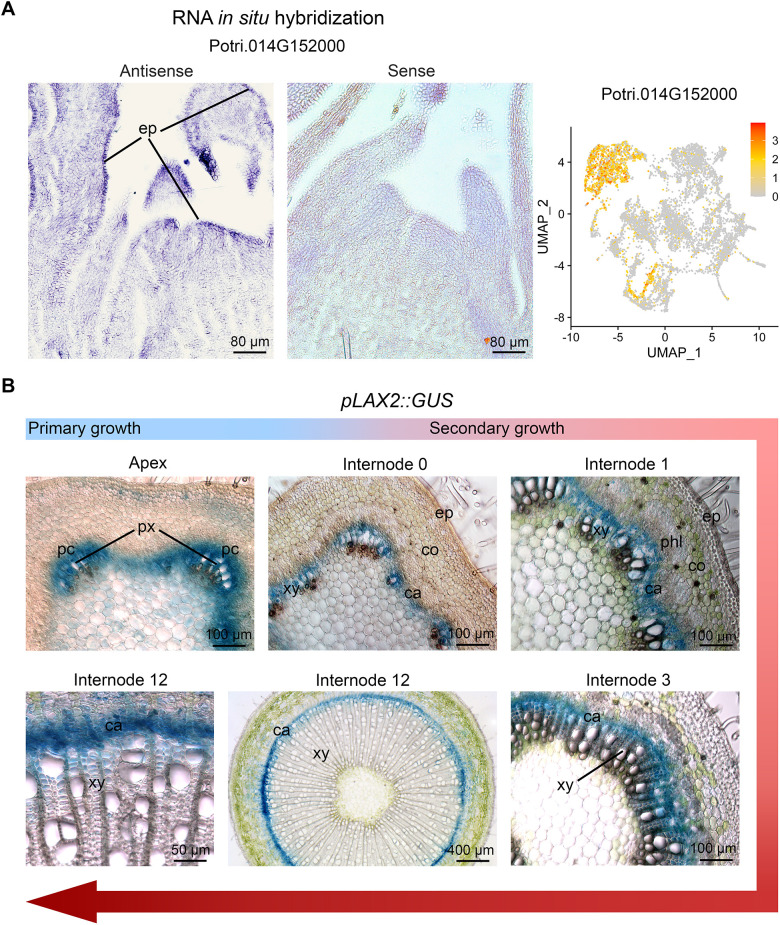
**Validation of cell type identity by RNA *in situ* hybridization and *promoter::GUS* fusions.** (A) RNA *in situ* hybridization shows that the expression of Potri.014G152000 is mainly located in the epidermis of the tissues present in the *Populus* shoot apex. (B) In stable *Populus* transgenic lines, GUS activity under the *LAX2* promoter confirmed that *LAX2* (blue) is expressed at the procambium and primary xylem during the early stages of vascular formation when the vasculature is still present in bundles in the stem. *LAX2* expression is restricted to the vascular cambium and the very first layers of the secondary xylem during the stem secondary growth. ca, vascular cambium; co, collenchyma; ep, epidermis; pc, procambium; phl, phloem; px, primary xylem; xy, xylem.

Our single-cell transcriptome analyses allowed us to infer the developmental trajectory of the epidermis, mesophyll and the primary vasculature in *Populus*. Epidermal cells are highly represented in our clustering (Fig. 1A). RNA *in situ* hybridization verified that the expression of a *de novo* identified marker for the epidermis, Potri.014G152000 (Table S3), enriched in this cell type at the apex (Fig. 5A). Moreover, vascular data integration identified regulatory genes potentially involved in the xylem, phloem and procambium development (Fig. 4E). We used a promoter fused to beta-glucuronidase (GUS) to assess the accuracy of the vasculature developmental trajectories inferred for the *Populus* apex. We generated stable transgenic lines to explore the *LAX2* (Potri.009G132100) promoter activity fused to GUS. A GUS staining assay showed that, as predicted by the single-cell data, *LAX2* is expressed at the procambium and primary xylem during the early stages of vascular formation in the apex, when the vasculature is still present in bundles (Fig. 5B). Further in the stem secondary growth, *LAX2* expression is restricted to the vascular cambium and the first layers of the secondary xylem (Fig. 5B). These results highlight the accuracy of cell clustering and annotation and the developmental trajectories identified.

### Characterization of *Populus* auxin influx carrier LAX2-like during xylem differentiation

Auxins are exported and imported from cell to cell using efflux and influx carriers: PIN and AUX/LAX, respectively. Auxin homeostasis is crucial to achieving the proper growth, and auxin transport is essential for auxin homeostasis along the whole plant body (Woodward and Bartel, 2005). In *Arabidopsis*, auxin influx carriers comprise a family containing four genes: *AUX1* and the *AUX1*-like genes *LAX1*, *LAX2* and *LAX3*, which share 75-80% similarity at the protein level. In *Arabidopsis*, all these members are expressed in the inflorescence shoot vascular tissues. *AUX1* and *LAX1* are expressed in procambial cells and protoxylem, *LAX2* in procambial cells and *LAX3* in procambial and phloem cells. The quadruple *aux1 lax1 lax2 lax3* mutant develops fewer vascular bundles than the wild type (WT) (Fàbregas et al., 2015). Moreover, compared with the WT, the mutant shows a reduced differentiation of the interfascicular fiber cells and the xylem cells within the shoot vascular bundle (Fàbregas et al., 2015). These results indicate that auxin influx carriers promote xylem differentiation in the plant shoot. More recently, it has been reported that *lax2* mutants have increased xylem length (measured as the length of a straight line traced from the last procambium cell layer to the inner xylem cells facing the center of stem cross-sections) and number of xylem cell rows. These results highlight that xylem differentiation involves a tight regulation of local auxin homeostasis and signaling maxima in xylem precursor cells (Moreno-Piovano et al., 2017).

**Fig. 6.**
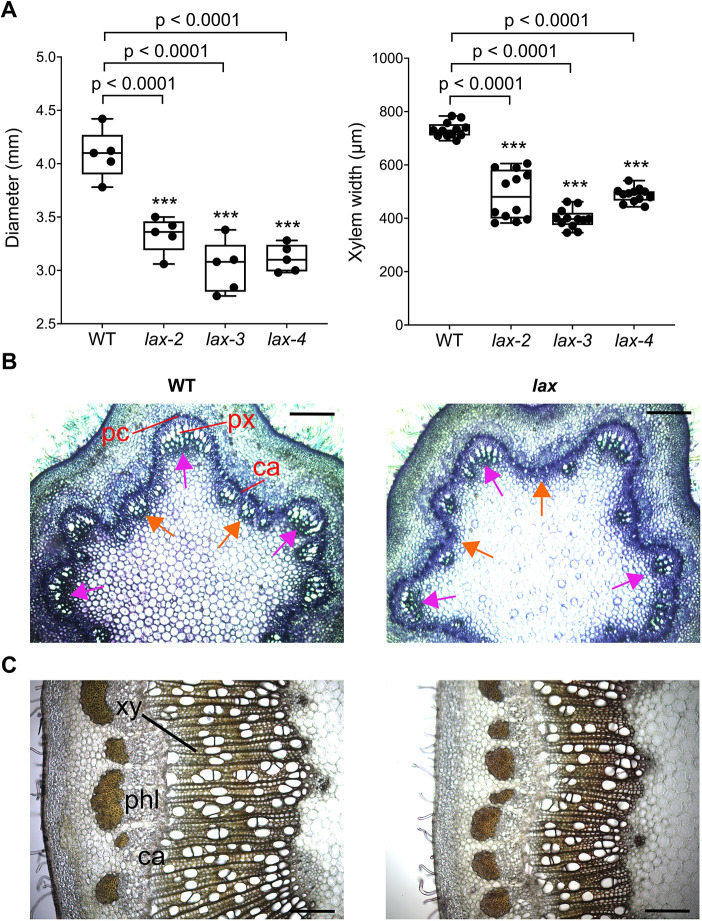
**Characterization of vascular development in lax mutants.** (A) After 6 weeks of growth, lax mutant plant diameter and xylem width was significantly reduced at internode 12 compared with WT. For each box-and-whisker plot: the center black line represents the median; the box extends from the 25th to 75th percentiles; the whiskers are the maximum and the minimum values (*n*=5 plants per each genotype for the diameter and 12 stem cross-sections from four plants per genotype for the xylem width). One-way ANOVA followed by Dunnett's test determined statistical differences between the WT and the mutants. ***P<0.001. (B) Stem cross-sections of internode 0 of WT and mutant trees. Cross-sections were stained with Toluidine blue O. Vascular bundles of the primary vasculature (pink arrows) showed a larger number of xylem tracheary elements cells in the WT. Moreover, the number of tracheary elements coming from the interfascicular cambium is also larger in the WT (orange arrows), suggesting a more differentiated xylem from the newly formed vascular cambium that connects vascular bundles. (C) Stem cross-sections of internode 12 of WT and mutant trees, stained with Phloroglucinol to highlight the presence of lignin. Mutant plants showed a significant reduction in the secondary xylem formation. ca, vascular cambium; pc, procambium; phl, phloem; px, primary xylem; xy, xylem. Scale bars: 200 µm in B; 100 µm in C.

In *Populus*, influx carriers remain uncharacterized. Our single-cell data analyses and promoter-GUS fusions showed that *Populus* LAX2-like gene expression resembles the expression of the *Arabidopsis AUX1* and *LAX1*, expressed in procambium/cambium and xylem cells (Fig. 5B). Moreover, the two *Populus* copies of *LAX2* were the only genes of the family with a very specific expression in the vascular domain (Fig. S9). Motivated by these observations, we characterized the role of *LAX2*-like genes during the vascular development in *Populus*. We generated CRISPR/Cas9 homozygous mutants for the two *Populus* LAX2 (Potri.009G132100 and Potri.004G172800) simultaneously. Three independent lines (*lax-2*, *lax-3* and *lax-4*), with mutations that generated truncated or altered versions of the proteins (Fig. S10), were selected for phenotypic characterization. Five WT plants and five plants per mutant line were grown in a growth chamber for 6 weeks. Mutants did not display aberrant or major developmental disruptions. However, the tree diameter was significantly reduced in the mutants (*P*-value adjusted≤0.001) (Fig. 6A). To compare the vascular development of mutants and WT plants at different stages in the stem, we examined the internodes 0 and 12. At the internode 0, xylem showed fewer tracheary elements in mutants compared with the WT. Moreover, the presence of tracheary elements was more evident in the WT than the mutants at the interfascicular vascular cambium, indicating more xylem differentiation in the vascular cambium that is already connecting the vascular bundles of the primary vasculature (Fig. 6B). At internode 12, mutant plants significantly reduced secondary xylem formation (Fig. 6C). Together, these results suggest that *LAX2*-like genes of *Populus* play a role in promoting xylem differentiation.

## DISCUSSION

Single-cell RNA-sequencing is revolutionizing the characterization of cell types and the developmental trajectories of cell lineages. Here, we report the application of this technology to create a comprehensive single-cell gene expression atlas of the vegetative shoot apex of the woody perennial model species *Populus*.

Plant cells are surrounded by a cell wall, which differs in composition and thickness according to the species, tissue, developmental stage and environmental conditions. The cell wall creates a hurdle for single-cell analysis of plant tissues, requiring its removal through enzymatic digestion and protoplast generation. Certain plant cell types (e.g. TRI) resist protoplasting (Zhang et al., 2021). More importantly, the protoplasting procedure can lead to changes in the cell transcriptome, such as the induction of stress-related genes. Challenges to the isolation of individual plant cells are further increased when working with highly lignified tissues, such as *Populus* stem. This study used snRNA-seq to overcome these problems. We show that the isolation and analysis of individual nuclei detect diverse cell types and numbers of transcripts to enable their discrimination at a resolution comparable with whole cells in plants. Our results also suggest that nuclei isolation overcomes the limitation of exploring the specific transcriptome of TRI when using microfluidic approaches. The plant material used in the present study contains an abundant representation of these cells (Fig. S11). Moreover, we demonstrated that this procedure could be applied to lignified *Populus* stems (Conde et al., 2021). The reported transcriptomes of shoot apex procambial cells, in combination with the feasibility of our nuclei isolation protocol to explore the single-cell transcriptome in the stem, constitute an unpreceded opportunity to dissect the missing link between primary and secondary growth, including how cambium develops from procambium at the initiation of secondary growth.

By sequencing the transcriptome of individual cells, we identified a similar cell population structure to the one observed in the *Arabidopsis* shoot. We traced the developmental trajectories for *Populus* mesophyll, epidermis and vasculature. Motivated by the similar cell type structure found between both species, we applied an analytical pipeline to compare cellular developmental programs occurring during shoot development in annual and perennial model species. For this comparison, we initially focused on primary vasculature development. Naturally, concern has been raised about whether snRNA-seq adequately captures accurate and representative transcriptomic information. Several articles comparing datasets derived from both methods concluded that, although generally a higher median number of genes per cell and higher total expressed genes are found when using protoplasts, there is a very high correlation between datasets generated by both methods. For example, snRNA-seq reflects the observed root transcriptome generated by whole-tissue and protoplast single-cell analysis (Farmer et al., 2021). Furthermore, a growing body of literature indicates that snRNA-seq achieves results comparable with scRNA-seq (Bakken et al., 2018; Wu et al., 2019) and the feasibility of integrating datasets from cells and nuclei (Mereu et al., 2020). Recently, several studies have applied the same technology to characterize cell-type specific transcriptomes in *Populus* stem or secondary xylem, using protoplasts (Chen et al., 2021; Li et al., 2021). *Populus* apex and stem are expected to contain conserved expression patterns in common cell types (e.g. epidermis, xylem and phloem) despite an enrichment in primary vasculature in the former and secondary vascular system in the latter. We observed that genes identified in our study to be present in phloem and xylem mother cells, sieve elements, CC and epidermis, including *AIL5*, *LAX2*, *APL* or *SEOR1*, are also markers for the same cell types in the stem (Fig. S12; Chen et al., 2021). This observation highlights the accuracy of our cell type annotation and the feasibility of comparing datasets from cells and nuclei. Our *promoter::GUS* staining results demonstrate the accuracy of unsupervised cell clustering and trajectory inference analysis in the delineation of cell differentiation toward the vasculature development in the *Populus* and *Arabidopsis* integrated data. In the future, this analytical framework can be applied to uncover the degree of conservation between cellular developmental programs that originate tissues such as the epidermis and mesophyll. This approach can also discover genes showing divergent expression patterns between species, which may explain their evolutionary differences. For example, the same framework could be applied to compare cell fate determination and lateral organs and vascular tissue pattern formation in monocots and dicots or between gymnosperms and angiosperms.

Our results show the power of scRNA-seq technology to discover gene function in *Populus*. The cell type-specific genes revealed by scRNA-seq may help identify phenotypic changes that occur at specific tissues or cells after altering the expression of cell type-specific genes. Functional characterization of Populus *LAX2* genes showed that cell-specific and comprehensive gene expression patterns revealed by scRNA-seq provide a valuable guide for choosing the tissue we should closely examine, thereby markedly increasing the success rate of reverse genetics.

One limitation of scRNA-seq in plants is to assign the mRNA profile of a cell to its position within a tissue or organ. One possible solution to address this challenge is the use of spatial transcriptomics, which allows the characterization of gene expression in barcoded regions of individual tissue sections. Although this technology has not been extensively applied in plant tissues (Giacomello et al., 2017), when combined with scRNA-seq or snRNA-seq, it has the potential to answer a wide range of biological questions concerning positional information in plants. Finally, although the 10x Genomics Chromium system is one of the most cost-effective and time-saving methods, the sensitivity (i.e. the probability of capturing and converting a particular mRNA transcript present in a single cell into a cDNA molecule) of such methods in which the sequencing reads are restricted to 3′ end of the transcripts is reduced compared with ‘full-length’ scRNA-seq methods (Ziegenhain et al., 2017). As in *Arabidopsis* when using the same technology, our scRNA-seq is insufficient to cluster previously identified cell types (i.e. CLV3 domain) within the SAM, because of the absence of the detection of *CLV3* gene expression.

The presence of unannotated clusters is a common observation in single-cell transcriptome studies, as they may not include a sufficient number of cells to support identifying a specific cell type. In our study, the careful exploration of the unannotated clusters 7, 9 and 14 did not point to a clear and definitive cell type. These clusters may also include novel or rare cell types that have not yet been characterized in a woody perennial plant like *Populus*. Nonetheless, the dot plot of the well-characterized genes (Fig. 1B) suggests that they may be transitioning cells related to proliferating, epidermal and meristem cells.

In summary, we have generated a gene expression map of the vegetative shoot apex at single-cell resolution for the perennial model species *Populus*. The definition of cells in distinct layers and functional zones of the *Populus* shoot apex now offers researchers the opportunity to investigate, at unprecedented resolution, how stem cells differentiate into distinct cell types in the shoot of perennial species, and compare those mechanisms with the vast knowledge generated in the annual plants model species *Arabidopsis*, where the root phloem differentiation mechanisms have been finely dissected (Otero et al., 2022; Roszak et al., 2021). Moreover, our procedure and results constitute a new opportunity to investigate the missing link between secondary growth in perennials by comparing the specific transcriptome of procambium and vascular cambium.

## MATERIALS AND METHODS

### Plant material and growth conditions

Shoot tips and stem cuttings from *in vitro* grown hybrid poplar (*Populus tremula*×*alba* INRA clone 717 1B4) were used as explants for shoot multiplication. The explants were cut into small segments (10-15 mm long) and placed aseptically in Murashige and Skoog media with vitamins (PhytoTech Labs, M519) supplemented with 0.1 g/L myo-inositol (PhytoTech Labs, I703), 0.25 g/L MES (PhytoTech Labs, M825), 2% (w/v) sucrose (PhytoTech Labs, S391) and solidified with 0.8% agar (PhytoTech Labs, A296). The pH of the medium was adjusted to 5.8 with KOH before autoclaving. Explants were grown in a growth chamber under long-day conditions (16 h light/8 h dark), 22°C, 65% relative humidity and 100 μmol m^−2^ s^−1^ photosynthetic photon flux. After three weeks in culture, a 5 mm-long portion of the shoot apex from 20 plants was excised and leaves were removed, leaving only leaf primordia.

### Nuclei isolation from *Populus* apex for snRNA-seq

To perform nuclei isolation, we followed the protocol that we previously developed for *Populus* shoot apices (Conde et al., 2021). Briefly, 20 dissected shoot apices were placed on a glass plate with 200 μl of the Nuclei Isolation Buffer (NIB; Conde et al., 2021). Next, samples were chopped with a sterile razor blade for 2 min. This step was repeated twice with a 30 s interval in between. The homogenate was washed with 5 ml of NIB with 0.2 U/μl of Protector RNase Inhibitor from the glass plate into a 50 ml conical tube and incubated on a rocking shaker for 5 min of gentle horizontal shaking. Samples were then filtered through one layer of pre-wetted (using NIB) miracloth laid on top of a 50 ml conical tube placed on ice and tilted on its side. Next, samples were filtered through a pre-wetted (using NIB) 40 μm strainer laid on top of a 50 ml conical tube placed on ice, tilted on its side, and washed with an additional 1-2 ml of NIB. Samples were then centrifuged at 600 ***g*** for 5 min at 4°C. After centrifugation, the supernatant was carefully removed, and the pellet was resuspended in 4 ml of NIB Wash pipetting very gently with a 2 ml Pasteur pipette. This step was repeated twice, except that after the last centrifugation, the pellet was resuspended in 750 μl of NIB Wash and transferred to 5 ml test tubes. To minimize the contamination of organelles such as chloroplast and remove the debris that could potentially clog the 10x Genomics microfluidic chips, we sorted the nuclei using Fluorescence Activated Nuclei Sorting. A total of 40,000 DAPI+ nuclei were sorted with a total recovery volume of 67-70 μl.

### Single nuclei cDNA and library preparation

We loaded 20,000 nuclei to the 10x Genomics Chromium Controller to generate the snRNA-seq library, following the 10x Genomics Chromium Single Cell v3.1 protocol. The cDNA reaction was prepared as described previously (Conde et al., 2021).

### Sequencing

Before full-scale library sequencing, a preliminary run was performed in an Illumina iSeq (∼ 4 million reads) to evaluate the parameters that are indicative of the quality of a scRNA-seq library, including the estimated number of cells captured and the fraction of reads in cells (i.e. percentage of ambient or ‘leaked’ RNA). The sequencing was performed using a standard program: 28 bp (cell barcode and UMI) for read 1, 90 bp (cDNA) for read 2, 10 bp for the I7 Index and 10 bp for the I5 Index. The assessment of RNA leakage was derived from the profile of the relationship between UMI counts and cell barcodes detected (Conde et al., 2021). After the library was considered suitable for full-scale sequencing (reads in cells>50% and at least 3000 cells captured at low sequencing depth), we proceeded to sequence in an Illumina NovaSeq6000. We obtained 502 million reads mapped to the genome, which resulted in a sequencing saturation of 78.9% and an average of 59,475 reads per nucleus. Sequencing was performed at the Interdisciplinary Center for Biotechnology Research at the University of Florida (Gainesville, FL, USA).

### Cell clustering

Cell clustering was performed using Asc-Seurat v2.1 (Pereira et al., 2021). The parameters used for cell clustering, using all cells or re-clustering specific cell types, are described in Fig. S1.

### Identification of novel *Populus* cell type-specific markers and DEG in the clusters

Cluster-specific genes were identified by Asc-Seurat using the Wilcox test with the default parameters log(FoldChange) ≥0.25, adjusted *P*-value≤0.05 and expression of the gene in at least 10% of the cells of the cluster.

### Integration pipeline for *Arabidopsis* and *Populus* single-cell expression data

We integrated the vasculature data generated by the snRNA-seq of *Populus* shoot apex (Fig. 2B; Fig. S2A) with the single-cell gene expression data of the shoot apex vasculature of *Arabidopsis* (Zhang et al., 2021). The one-to-one homology mapping of *Populus trichocarpa* Torr. and Gray and *A. thaliana* genes was obtained from extensive gene orthology generated from protein sequence data from 93 species genomes/transcriptomes. The input protein sequences included those from *P. trichocarpa* v4.1 (Tuskan et al., 2006) and *A. thaliana* Araport 11 (Cheng et al., 2017) genome assemblies, respectively. Gene ortho-groups and resolved (reconciled) gene trees were generated using the OrthoFinder 2 (Emms and Kelly, 2019) framework, based on the blastp (McGinnis and Madden, 2004) (v2.2.28) and MCL (Enright, 2002) (v14-137) methodologies for gene orthology inference. The resolved gene trees from OrthoFinder 2 were further parsed by last common ancestor (LCA) duplication events with respect to the tree of 93 species, to highlight better recent evolutionary dynamics across the species of interest in a refined set of parsed ortho-groups. For each parsed ortho-group (sub-tree), we assessed whether there was one gene member from both *P. trichocarpa* and *A. thaliana*, and identified such pairs of genes as one-to-one mappings. This analysis resulted in 9827 high-confidence mappings for subsequent analysis of the data presented in this work. The data integration was performed using Seurat, making use of the expression of these 9827 genes, in addition to the expression of 15 well-known tissue-specific markers showing a specific expression pattern in our vasculature single-cell data (Table S5).

### RNA *in situ* hybridization

For probe synthesis, a 333 bp PCR fragment amplified from cDNA using the primers 5′-GCTCTTTCTCTTTCCATGGCAA-3′ and 5′-TATCTTGTGGAAAAGAGAGCAG-3′ was cloned into pGEM T-easy (Promega). The probe was designed at the 5′ untranslated region of the Potri.014G152000 transcript. The probes were *in vitro* transcribed to obtain digoxigenin-UTP (DIG)-labeled RNA probes using T7 and Sp6 RNA polymerases for the sense and the anti-sense mRNA probes, respectively, using a DIG RNA Labeling Kit following the manufacturer's protocol (Roche). RNA probes were then purified by precipitation with an equal volume of 4 M NH_4_Ac and two volumes ethanol at −20°C overnight. *Populus* apexes from plants growing at the greenhouse for 4 weeks (under a long-day regimen at 22°C, 65% relative humidity and 100 μmol m^−2^ s^−1^ photosynthetic photon flux) were fixed, paraffin-embedded and sectioned as previously described (Conde et al., 2017). The hybridization protocol was performed as described in http://plantlab.caltech.edu/html/protocols.html. The final probe concentration was 0.5 ng/µl and the hybridization was performed at 55°C overnight. For detection of hybridization signals, a DIG Nucleic Acid Detection Kit (Roche) was used following the manufacturer's protocol with minor modifications: the antibody was diluted to 1:1250, and three washing steps of 15 min each were performed. The images were obtained using a Zeiss Axioplan 2 microscope attached to a QImaging Retiga EXi Fast 1394 camera.

### Validation of the predicted marker genes by GUS staining analysis

#### Cloning

For cloning the DNA constructs used in the present study, the Golden Gate system was used (Engler et al., 2014). A 1715 bp segment of the promoter (up to the ATG start codon) of hybrid poplar *LAX2* gene was amplified by PCR. The 4-nucleotide overhangs and the BsaI target sites at 5′ strands at both borders were added by including these sequences in the primers. The promoter sequence was cloned in frame with the beta-glucuronidase (GUS) CDS (pICH75111; MoClo Plant Parts Kit) and the 35S terminator into the cloning vector (pICH47811; MoClo Toolkit) by using the level 1 reaction.

To characterize *pLAX2::GUS* fusion activity, we generated stable transgenic lines. To identify the positive events of the transformation, we used the hygromycin resistance genes under the Atact2 promoter (pICH87644; MoClo Plant Parts Kit) cloned in the pICH47802 vector (MoClo Toolkit). Both transcriptional units containing the marker gene for the transformation and GUS, respectively, were cloned together into the final expression vector pAGM4673 (MoClo Toolkit) using the Golden Gate level 2 reaction. *Agrobacterium tumefaciens*-mediated transformation was performed using the strain GUV3101 in the *Populus tremula*×*alba* 717-1B4 genotype, using the protocol described in Gallardo et al. (1999).

#### GUS staining and preparation of cross-sections

Three-week-old *in vitro* transgenic plants containing the *pLAX2::GUS* construct were transferred to pots as described in Ribeiro et al. (2020). After 4 weeks of growth under a long-day regimen at 22°C, 65% relative humidity and 100 μmol m^−2^ s^−1^ photosynthetic photon flux, a 5 mm-long portion of the apex, internode 1, 3 and 12, were transferred to the GUS staining solution [5 nM potassium ferrocyanide, 5 nM potassium ferricyanide, 0.1 M sodium phosphate buffer, 1 mM sodium EDTA, 1% Triton X and 0.3% X-Gluc (previously dissolved in *N*,*N*-dimethylformamide)].

Apices and stems were incubated for 2-4 h in the solution. Next, plant material was fixed in 4% formaldehyde as previously described (Conde et al., 2013). Apices and stems were embedded in 6% (w/v) agarose, and 40-60 µm thick cross-sections were obtained in a Leica VT1000S vibratome. The images were obtained using a Zeiss Axioplan 2 microscope attached to a QImaging Retiga EXi Fast 1394 camera. To determine the localization of the GUS during the primary and secondary growth phases of stem development, we analyzed stem segments in the apex and several internodes below the apex [leaf plastochron indices or internodes 0, 1, 3 and 12, where internode 1 refers to the stem portion between the first and the second fully expanded leaves counting from the apex to the bottom (Dharmawardhana et al., 2010; Larson and Isebrands, 1971)].

### Functional characterization of LAX-like genes in *Populus*

#### Cloning

Hybrid poplar CRISPR/Cas9-mediated homozygous mutants for *LAX2* were generated expressing two single guide RNAs (sgRNAs), targeting the two *Populus* paralog genes coding sequence (Fig. S9). The DNA constructs were generated using the Golden Gate-based system that we recently designed for multi-site genome editing in hybrid poplar (Triozzi et al., 2021). *Agrobacterium*-mediated transformation was performed using the strain GUV3101 in the *Populus tremula×alba* 717-1B4 genotype, as described above. Independent lines obtained by hygromycin selection were genetically screened to identify the specific allele mutation as described before (Triozzi et al., 2021) (Fig. S10).

#### Characterization of the vasculature in the lax mutant trees.

To evaluate the vascular development in the lax mutants and WT plants during primary and secondary growth, three-week-old *in vitro* mutant and WT plants were transferred to pots as described above. After 6 weeks of growth under a long-day regimen at 22°C, 65% relative humidity and 100 μmol m^−2^ s^−1^ photosynthetic photon flux, internode 0 (immediately above the first full expanded leaf of the apex) and 12 were fixed in 4% formaldehyde. Internodes were embedded in 6% (w/v) agarose and 40-60 µm thick cross-sections were obtained in a Leica VT1000S vibratome. Internode 0 was stained with a 0.5% solution of Toluidine blue O in PBS. Internodes 12 were clarified for 5 min in 70% ethanol and stained with 2.5% phloroglucinol-HCl to observe the presence of lignin. The images were obtained using a Zeiss Axioplan 2 microscope attached to a QImaging Retiga EXi Fast 1394 camera. Five WT plants and five plants per each of the three mutant lines were used for the phenotypical measurements. Three vibratome stem cross-sections per internode from four WT plants and from four plants per mutant line were measured to calculate the xylem width, using the ImageJ software.

## Supplementary Material

10.1242/develop.200632_sup1 Supplementary information
