# Supplementary material for: Single-nuclei transcriptome analysis of the shoot apex vascular system differentiation in Populus
Source: Development. 2022 Oct 17;149(21):dev200632. doi: 10.1242/dev.200632 (PMC9720752; doi:10.1242/dev.200632)
Supplement: Supplementary information [file develop-149-200632-s1.pdf]

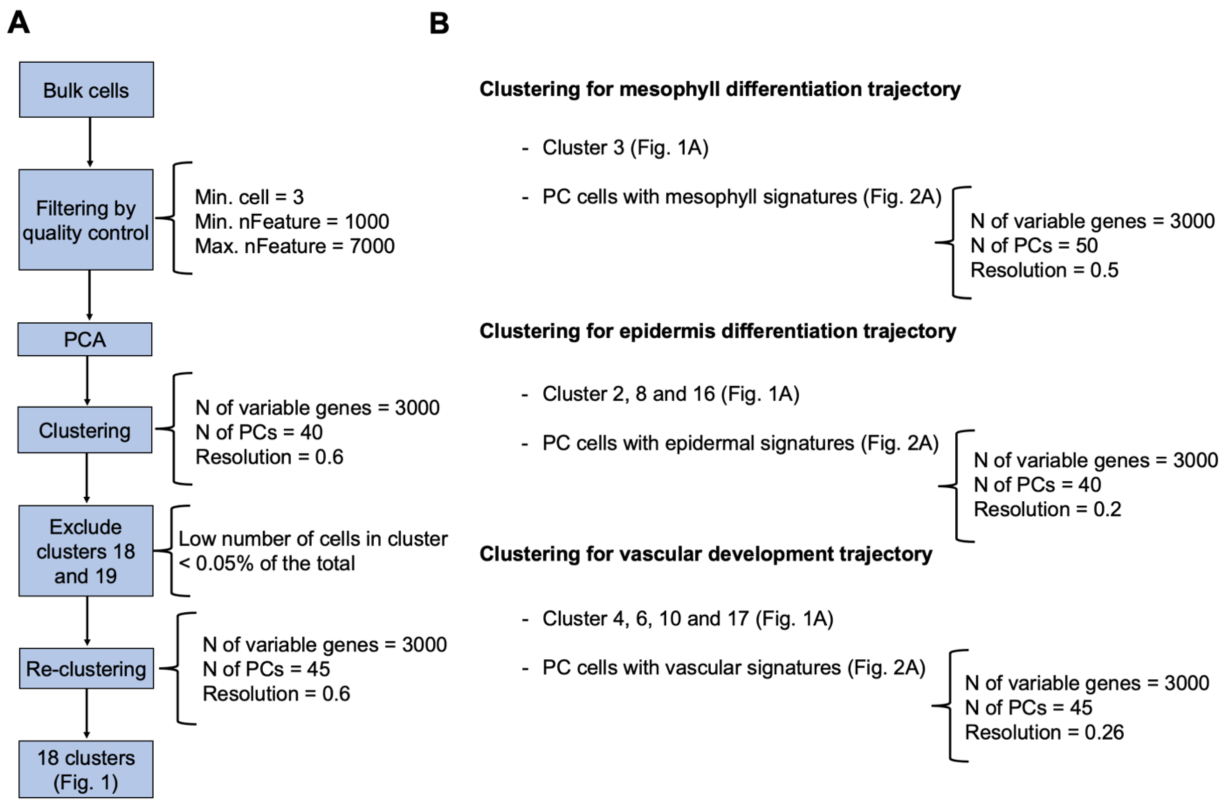

Fig. S1. (A) Workflow followed in Asc-Seurat to cluster the individual nuclei transcriptomes obtained from the hybrid poplar vegetative shoot apex. **(B)** The mesophyll, epidermis, and vascular cells were re-clustered together with their corresponding proliferating cells (PC), following the parameters shown in this figure.

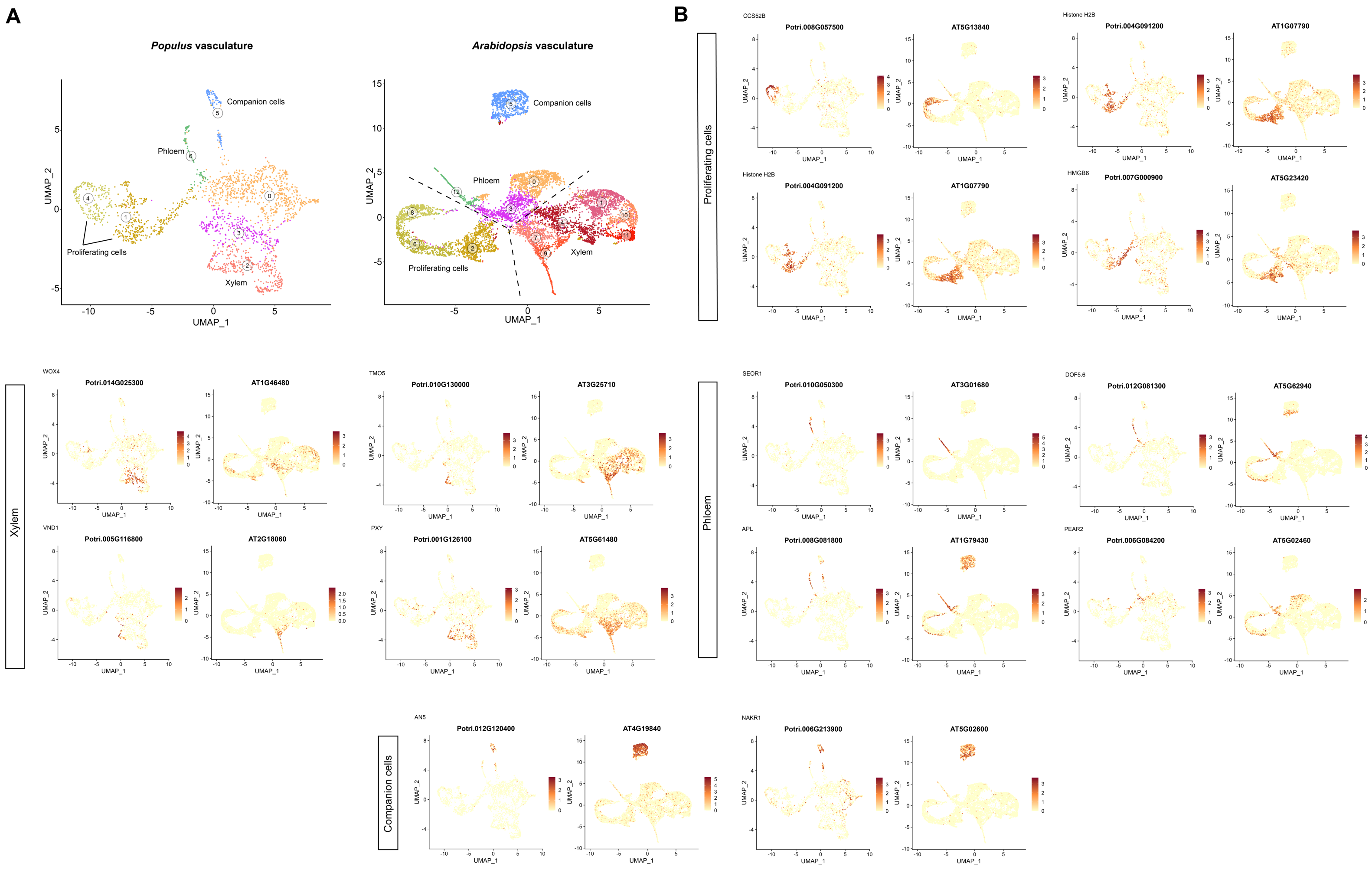

Fig. S2. (A) Visualization of the *Populus* and *Arabidopsis* vascular tissue cell populations with the proliferating vascular cells by UMAP. Dots, individual cells; color, cell clusters. **(B)** Expression profiles of well-known markers identified proliferating cells, xylem, and companion cells.

Development • Supplementary information

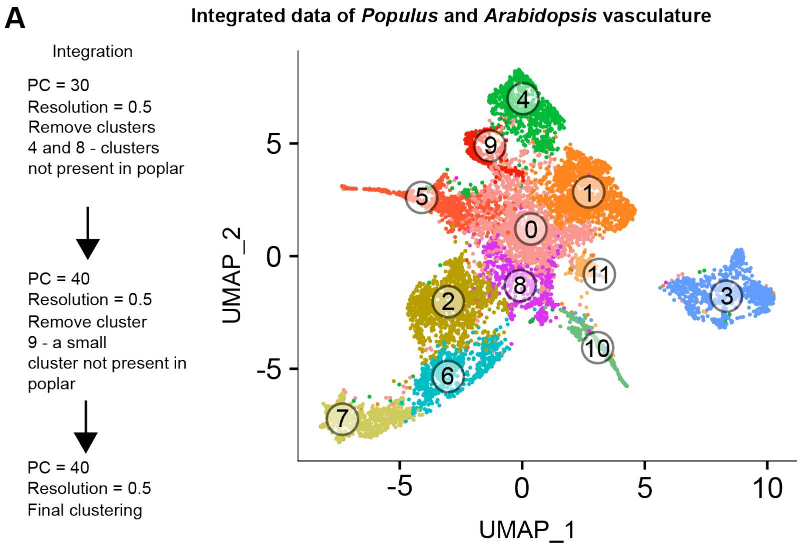

B Number of cells en each cluster

Cluster	*Populus*	*Arabidopsis*
0	195	1227
1	687	572
2	333	777
3	88	732
4	99	635
5	100	508
6	124	479
7	102	483
8	160	280
9	115	162
10	55	172
11	23	104

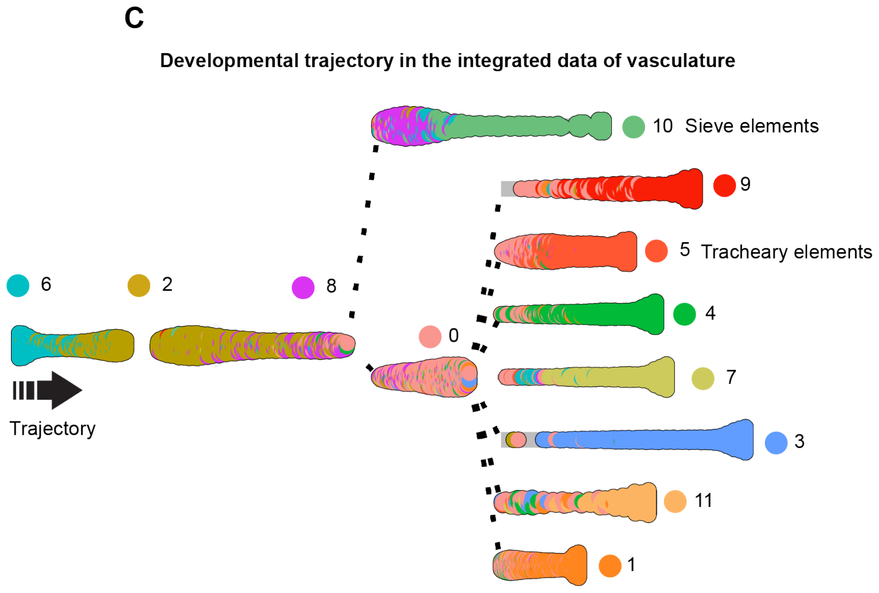

Fig. S3. (A) Workflow followed in Asc-Seurat to cluster the cells after the *Populus-Arabidopsis* integration of the apex vasculature data. **(B)** the number of cells identified in each cluster for *Populus* and *Arabidopsis* after the data integration. **(C)** Slingshot was used to generate the overall trajectory for vasculature in the *Populus-Arabidopsis* integrated data to identify the clusters involved in the sieve and tracheary elements differentiation.

Development • Supplementary information

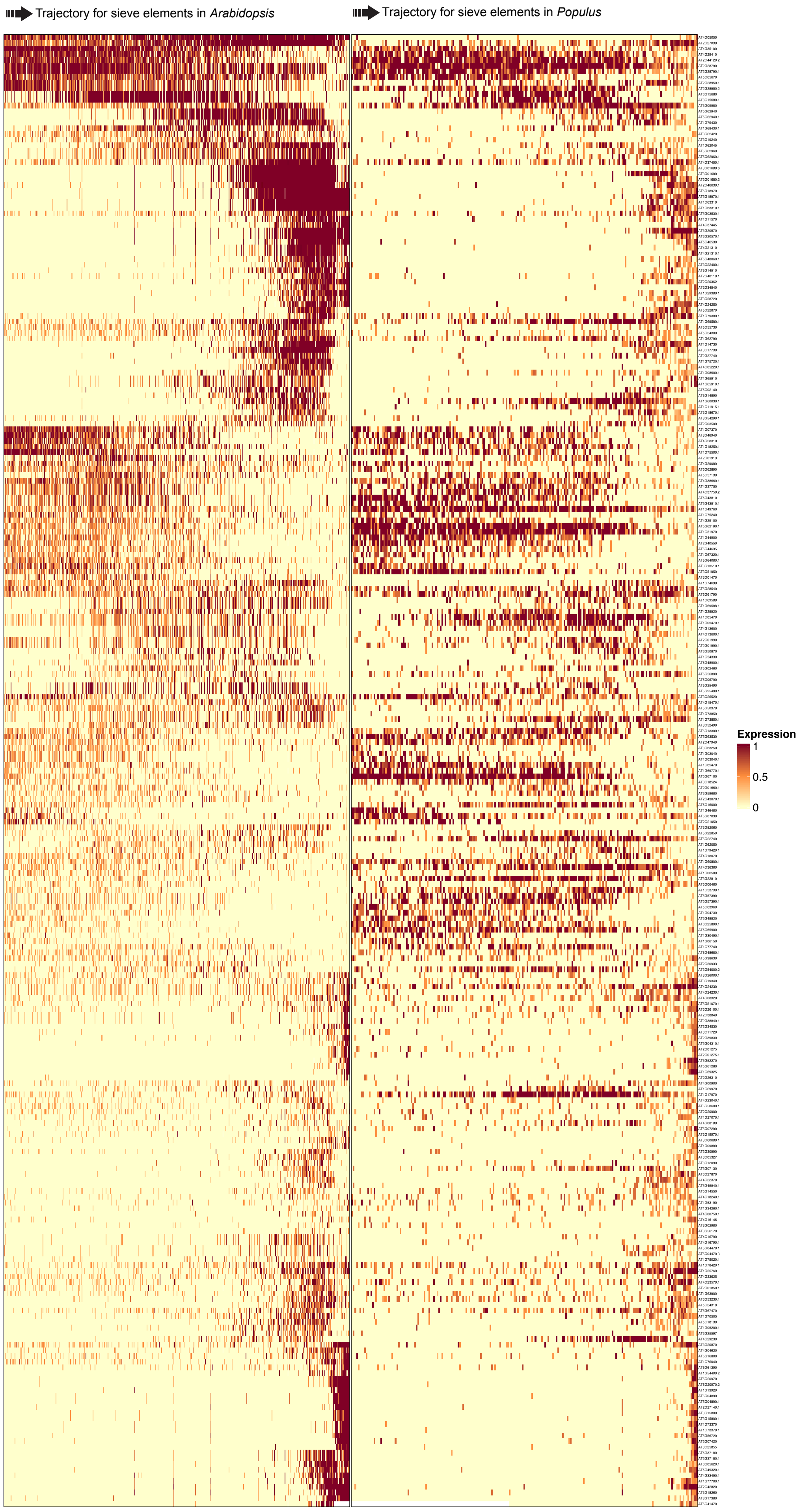

Fig. S4. Heatmap showing the expression of the differentially expressed genes in the developmental trajectories of the sieve elements, common in both *Populus* and *Arabidopsis* species, at single-cell resolution along the trajectory.

Development • Supplementary information

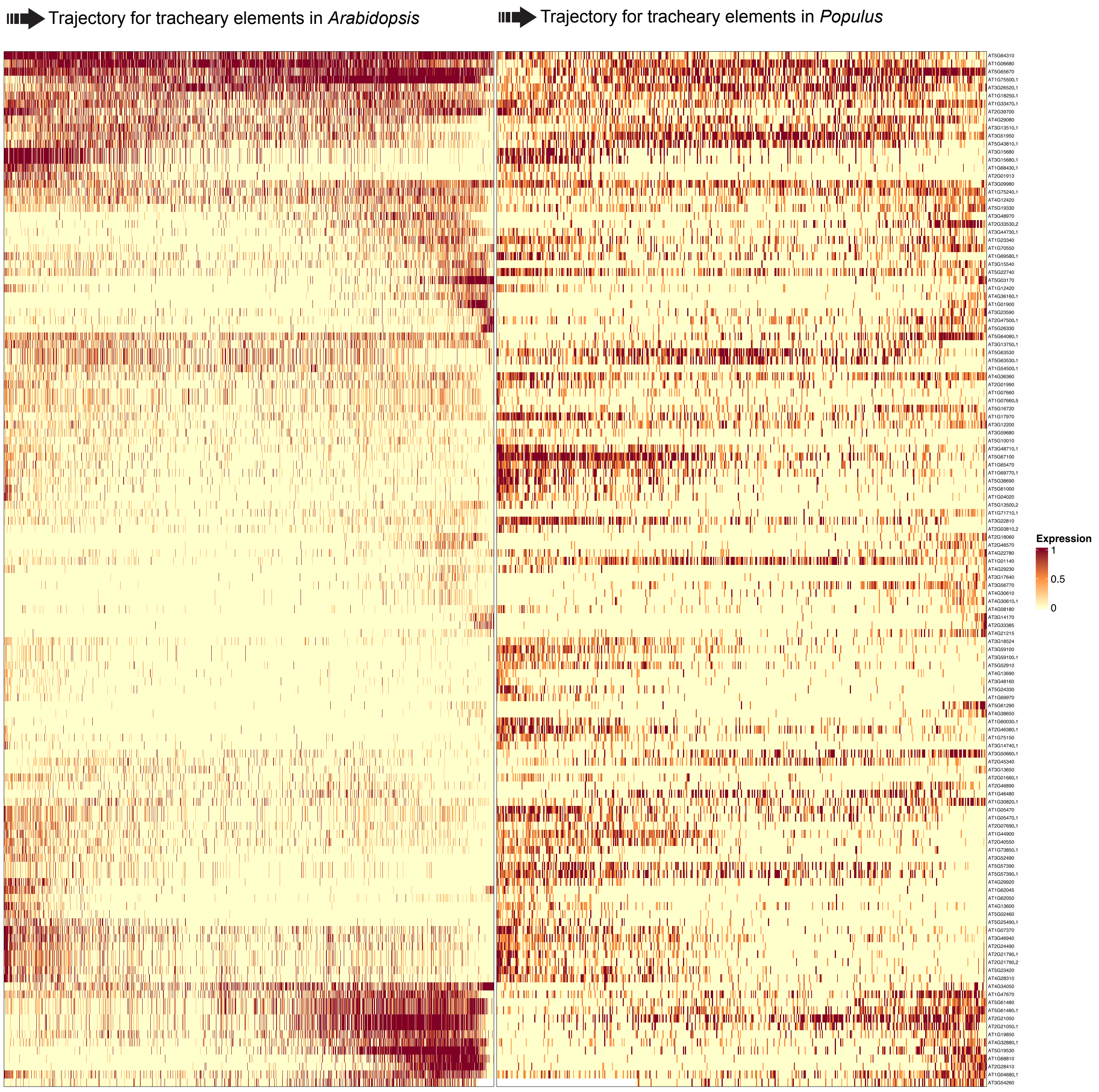

Fig. S5. Heatmap showing the expression of the differentially expressed genes in the developmental trajectories of the tracheary elements, common in both *Populus* and *Arabidopsis* species, at single-cell level resolution along the trajectory.

Development • Supplementary information

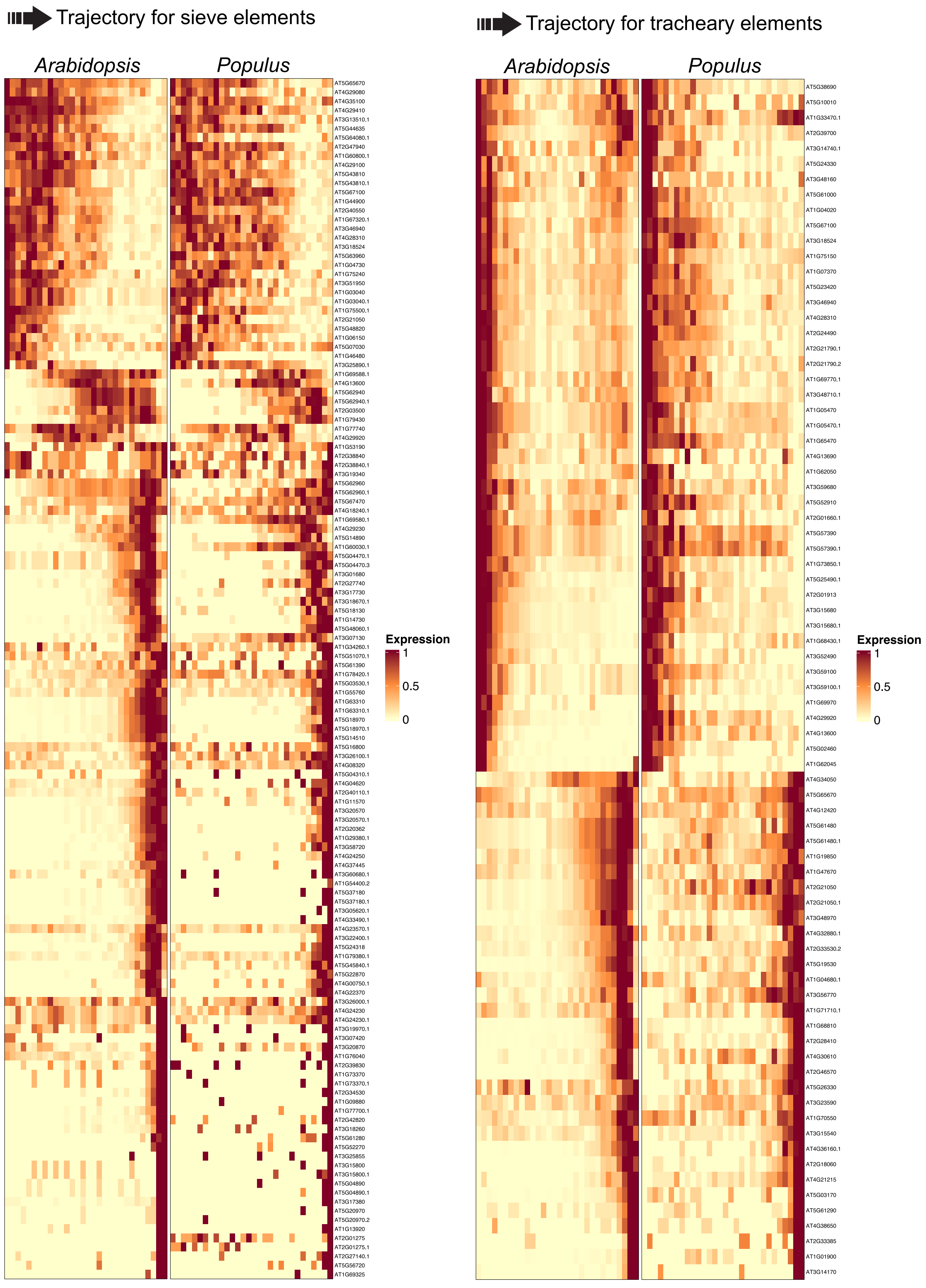

Fig. S6. Heatmap showing the expression of the differentially expressed genes in the developmental trajectories of sieve and tracheary elements in *Populus* and *Arabidopsis*, with the same expression pattern in both species based on their positive correlated expression (Corr. ≥ 0.5; FDR ≤ 0.01), calculated by dividing the trajectory into 30 bins.

Development • Supplementary information

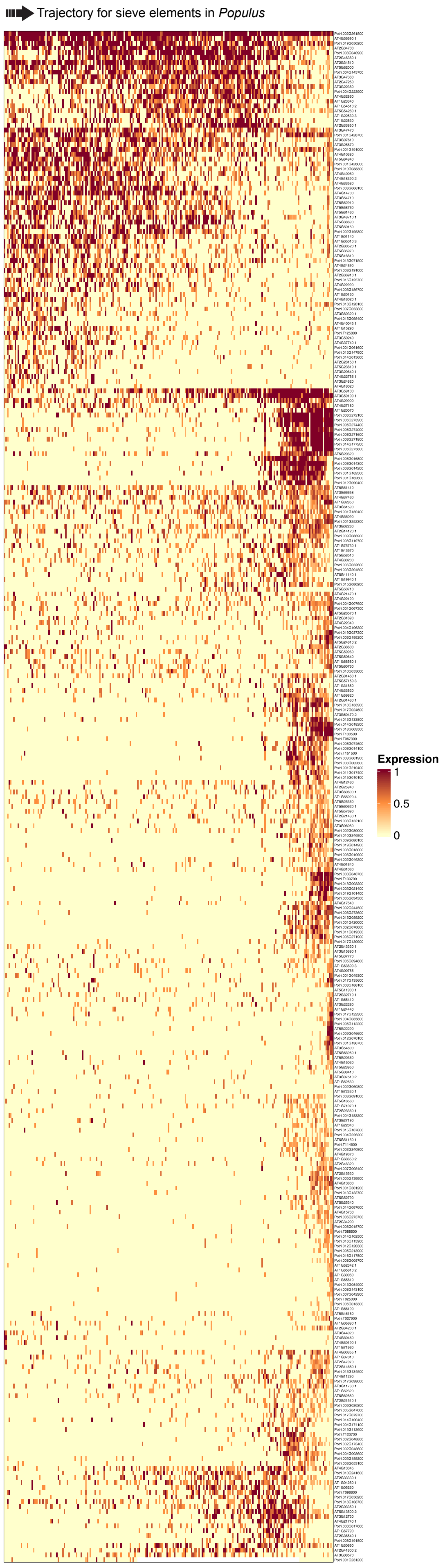

Fig. S7. Heatmap showing the expression of the differentially expressed genes in the sieve elements developmental trajectory only in *Populus*, at single-cell level resolution along the trajectory.

Development • Supplementary information

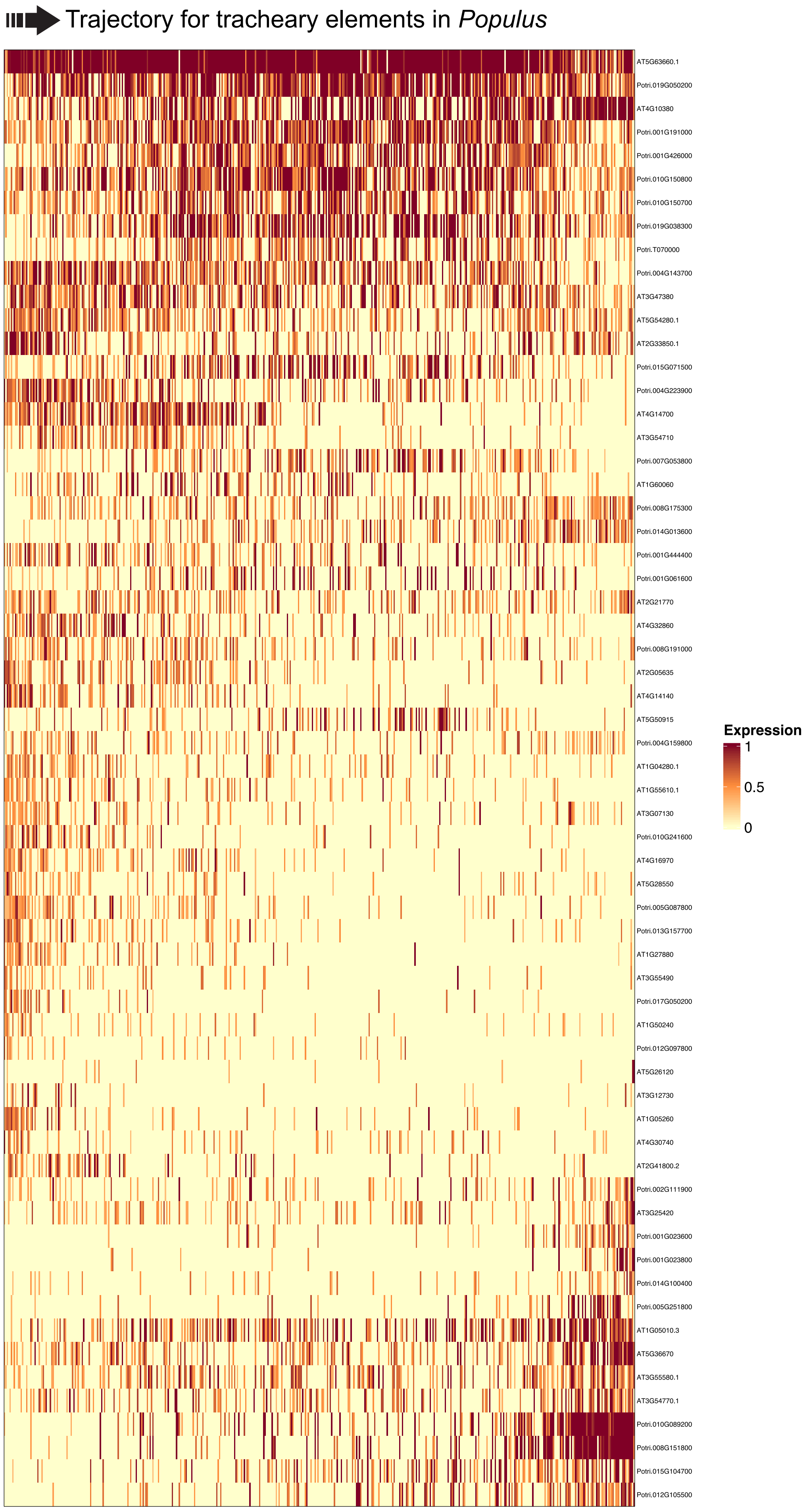

Fig. S8. Heatmap showing the expression of the differentially expressed genes in the tracheary elements developmental trajectories only in *Populus*, at single-cell level resolution along the trajectory.

Development • Supplementary information

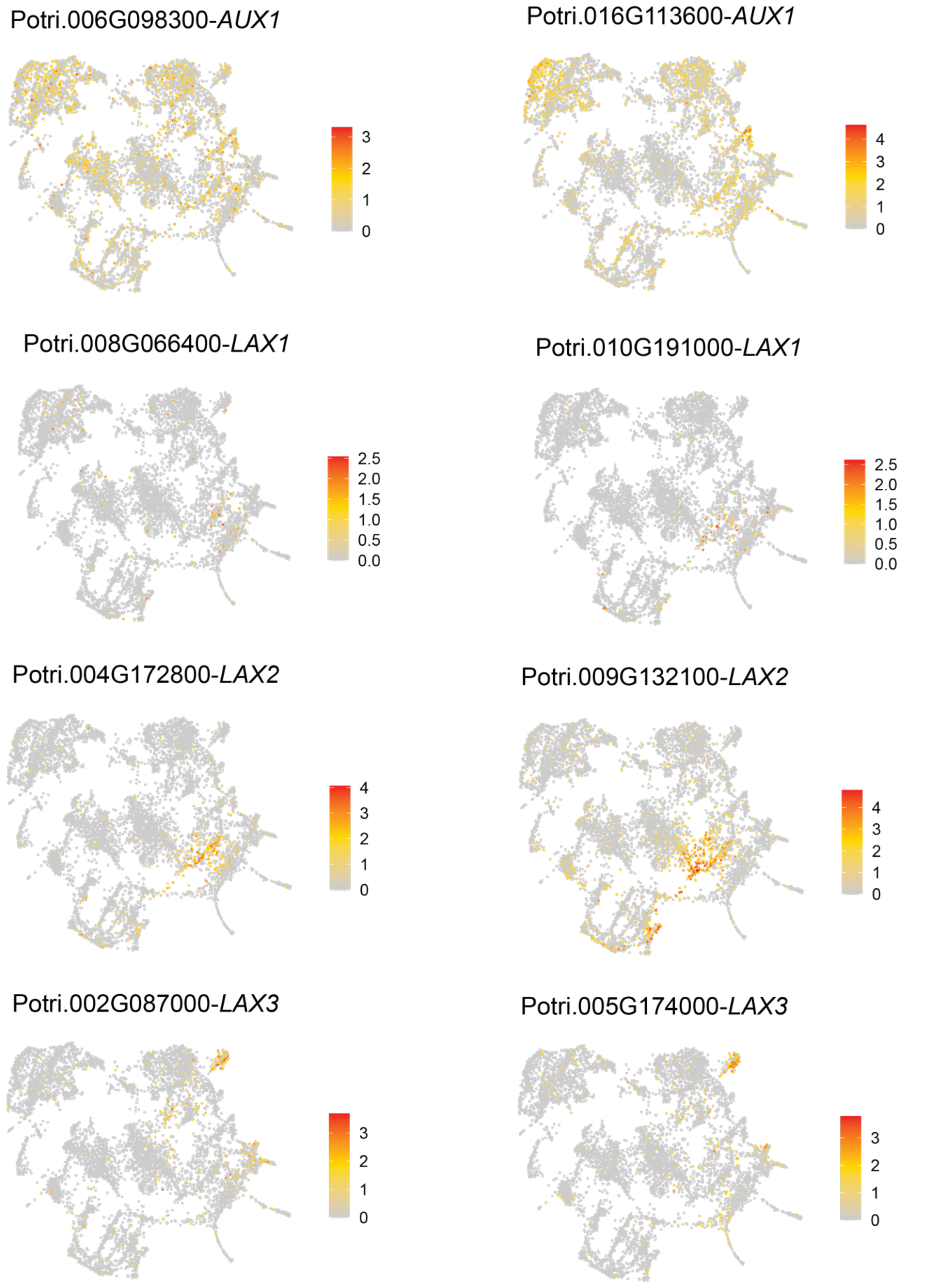

Fig. S9. Expression of the *Populus* auxin influx carriers in the vegetative shoot apex cell population.

Development • Supplementary information

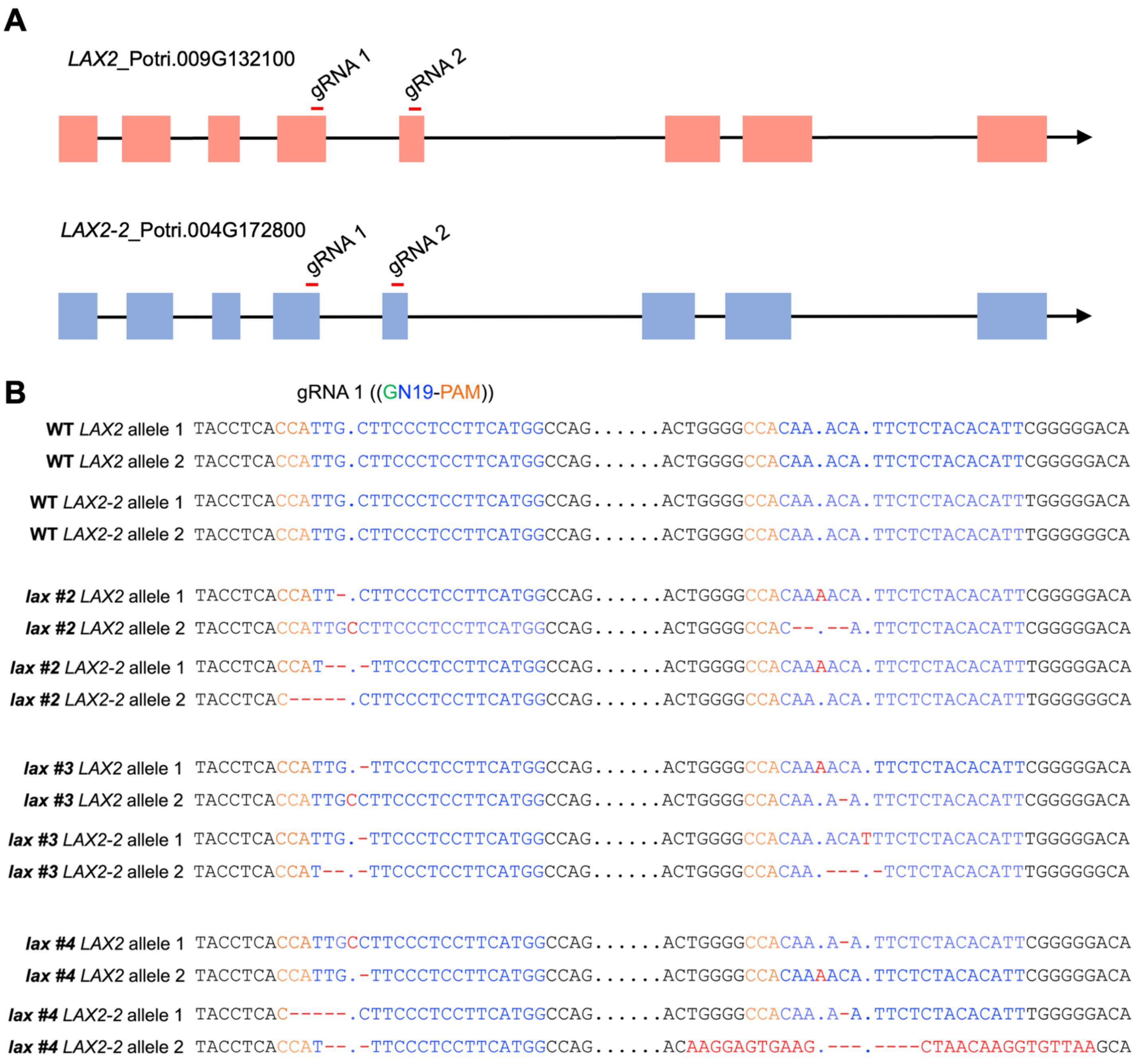

Fig. S10. CRISPR/Cas9 mutations of *LAX2*-like genes in *Populus tremula* × *alba* INRA clone 717 1B4. **(A)** *Agrobacterium tumefaciens*-mediated transformation generated transgenic lines expressing Cas9 nuclease and two guide RNAs (gRNAs). Each gRNA contains a sequence of 19 nucleotides (N19) that binds to the target DNA, guiding Cas9 to the two copies of *LAX2* CDS sites (fourth and fifth exons). The protospacer adjacent motif (PAM, NGG, orange) is located at 3' downstream of the target sequence and is necessary for the Cas9 to cleave the target site to generate a double-strand break (DSB). When a DSB was repaired, various mistakes were created in or near the target locus, such as small insertions and deletions. **(B)** Three independent lines (*lax-2, lax-3* and *lax-4*) were obtained, containing mutations in both alleles in each of the two copies of *LAX2*-like genes present in *Populus*.

Development • Supplementary information

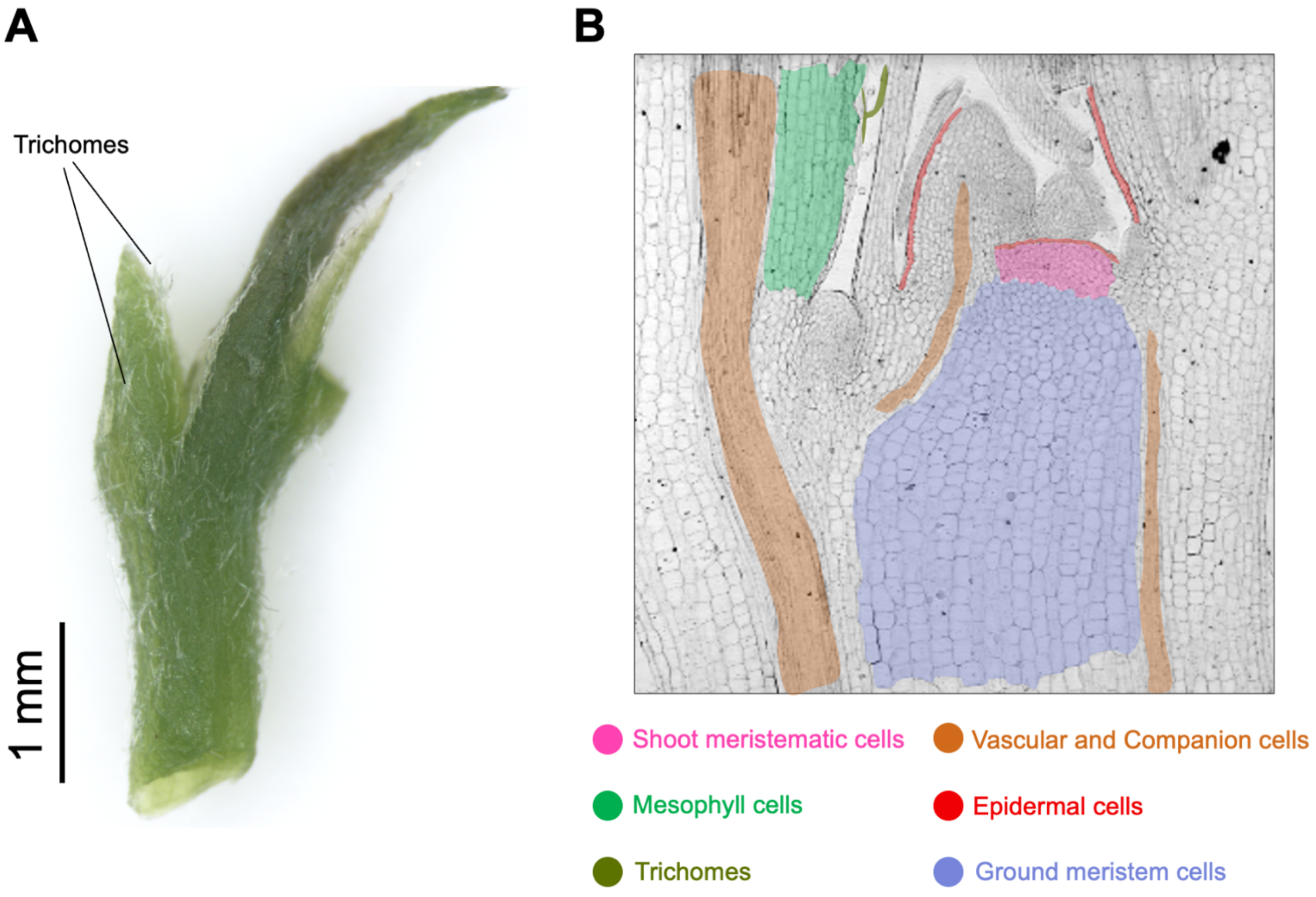

Fig. S11. (A) Image of a *Populus* apex as the ones used in the present study for nuclei isolation (see Materials and Methods). **(B)** Longitudinal section of a *Populus* apex. Colors represent the cell type identified in the present study.

Development • Supplementary information

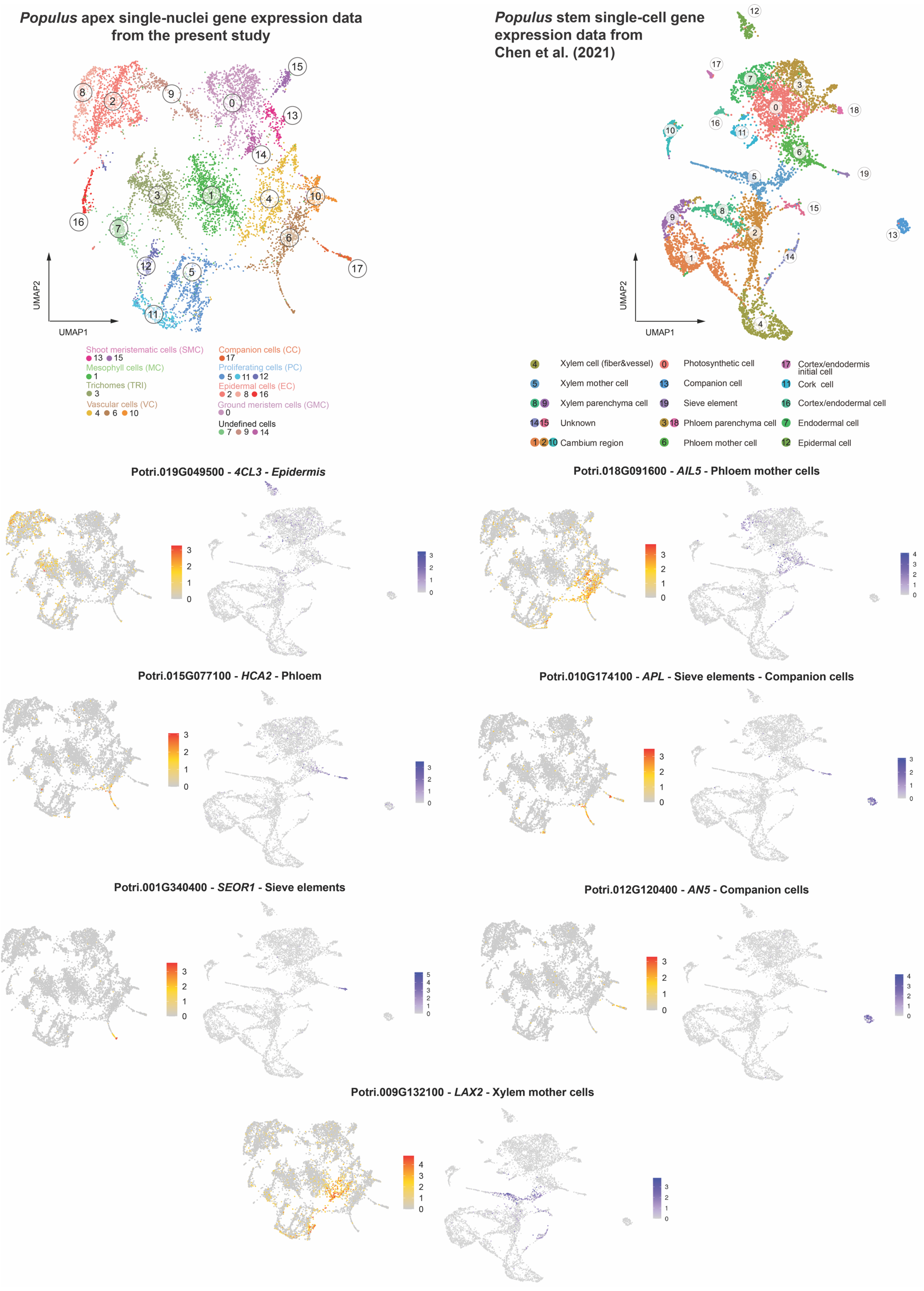

Fig. S12. Comparison between the gene expression pattern of markers for epidermis, phloem, companion cells, and xylem identified in the *Populus* shoot apex cell population in the present work, and the *Populus* stem cell population identified in Chen et al. (221). These genes are also markers for the same cell type in the stem, pointing to the accuracy of our clustering annotation. To explore stem data, we used the web server developed by Chen and colleagues (https://scu-populus.shinyapps.io/scRNAPal/).

Development • Supplementary information

Table S1. Summary of snRNA sequencing and quality control overview.

**Poplar apex**	
Number of Nuclei	8,324
Total Number of Genes Detected	31,214
Mean Genes per Nucleus	2,477
Median Genes per Nucleus	2,308
Mean UMIs per Nucleus	3,618
Median UMIs per Nucleus	3,206
Mean UMIs per Gene	965
Median UMIs per Gene	408

Table S2. List of Populus cell type-specific markers, generated by evaluating the expression of poplar homologous to Arabidopsis markers for the different domains of the vegetative or reproductive shoot apex, looking for a significant accumulation of their transcripts in any of the 18 clusters identified in Populus. We also evaluated the expression of homologous genes that in Arabidopsis are mainly expressed at different cell types of the vegetative and the reproductive shoot apex. Moreover, we identified all the cell markers for each cluster identified in the poplar shoot apex (Table S3) and identified those whose biological functions or expression patterns have been well studied. Based on these three levels of information, we generated this table containing the Populus tissue specific markers used during clustering annotation.

Click here to download Table S2

Table S3. De novo identification of cell-type marker genes in Populus. Asc-Seurat application was used to identify marker genes for each cluster. Markers were defined as genes significantly induced in the corresponding cluster. Gene annotation was performed following the file containing Populus homologs to Arabidopsis, named Ptrichocarpa_533_v4.1.annotation_info.txt available at Phytozome. TAIR 10 was used for gene annotation.

Click here to download Table S3

Table S4. GO enrichment analysis for the gene markers of cluster 1. Arabidopsis gene IDs were used in the AGRIGOv2 website (http://systemsbiology.cau.edu.cn/agriGOv2) to identify the gene ontologies significantly overrepresented in cluster 1, using the TAIR10 annotation.

Click here to download Table S4

Table S5. Set of 9,842 Arabidopsis and Populus one-to-one homolog genes used to integrate the Arabidopsis and Populus primary vasculature single-cell data.

Click here to download Table S5

Table S6. Complete list of Populus-Arabidopsis homolog genes created by combining our phylogenomic pipeline and the Populus annotation information available at Phytozome. After the integration, the Arabidopsis IDs are used to check the expression of Arabidopsis and Populus genes. To differentiate between multiple copies of the same gene present in Populus, a dot followed by a number was added to the Arabidopsis gene IDs.

Click here to download Table S6

Development • Supplementary information

Table S7. List of the 259 genes, significantly (FDR ≤ 0.01) associated with the phloem differentiation trajectory, in Arabidopsis and Populus, identified by tradeSeq model. Genes were annotated based on Tair 10.

Click here to download Table S7

Table S8. List of the 129 genes, significantly (FDR ≤ 0.01) associated with the xylem differentiation trajectory, in Arabidopsis and Populus, identified by tradeSeq model. Genes were annotated based on Tair 10.

Click here to download Table S8

Table S9. Set of 132 genes with a robust gene expression correlation (Corr. ≥ 0.5; FDR ≤ 0.01) in both species Arabidopsis and Populus, in the developmental trajectory for the phloem differentiation.

Click here to download Table S9

Table S10. Set of 78 genes with a robust gene expression correlation (Corr. ≥ 0.5; FDR ≤ 0.01) in both species Arabidopsis and Populus, in the developmental trajectory for the xylem differentiation.

Click here to download Table S10

Table S11. List of the genes, significantly (FDR ≤ 0.01) associated with the phloem differentiation trajectory, specifically for Populus, identified by tradeSeq model. Genes were annotated based on Tair 10.

Click here to download Table S11

Table S12. List of the genes, significantly (FDR ≤ 0.01) associated with the xylem differentiation trajectory, specifically for Populus, identified by tradeSeq model. Genes were annotated based on Tair 10.

Click here to download Table S12

Table S13. Set of Populus genes significantly induced (FDR < 0.05) in the cluster 8 of the integrated data.

Click here to download Table S13

Table S14. Set of Populus genes significantly induced (FDR < 0.05) in the cluster 0 of the integrated data.

Click here to download Table S14

Table S15. Set of Populus genes significantly induced (FDR < 0.05) in clusters 0 and 8, also induced in those clusters in Arabidopsis, after the Arabidopsis-Populus data integration.

Click here to download Table S15

Development • Supplementary information
