# Supplementary material for: Single-nuclei transcriptome analysis of the shoot apex vascular system differentiation in Populus
Source: Development. 2022 Oct 17;149(21):dev200632. doi: 10.1242/dev.200632 (PMC9720752; doi:10.1242/dev.200632)
Supplement: Supplementary file 1 [file develop-149-200632_review_history.pdf]

Single-nuclei transcriptome analysis of the shoot apex vascular system differentiation in *Populus*

Daniel Conde, Paolo M. Triozzi, Wendell J. Pereira, Henry W. Schmidt, Kelly M. Balmant, Sara A. Knaack, Arturo Redondo-López, Sushmita Roy, Christopher Dervinis and Matias Kirst

DOI: 10.1242/dev.200632

Editor: Yka Helariutta

Review timeline

Original submission:	8 February 2022
Editorial decision:	14 March 2022
First revision received:	8 August 2022
Editorial decision:	30 August 2022
Second revision received:	1 September 2022
Accepted:	20 September 2022

Original submission

First decision letter

MS ID#: DEVELOP/2022/200632

MS TITLE: Single-cell analysis of the shoot apex vascular system differentiation in Populus

AUTHORS: Daniel Conde, Paolo M. Triozzi, Wendell J. Pereira, Henry W. Schmidt, Kelly M. Balmant, Sara A. Knaack, Sushmita Roy, Christopher Dervinis, and Matias Kirst

I have now received all the referees' reports on the above manuscript, and have reached a decision. The referees' comments are appended below, or you can access them online: please go to BenchPress and click on the 'Manuscripts with Decisions' queue in the Author Area.

As you will see, the referees express considerable interest in your work, but have some significant criticisms and recommend a substantial revision of your manuscript before we can consider publication. If you are able to revise the manuscript along the lines suggested, which may involve further experiments, I will be happy receive a revised version of the manuscript. Your revised paper will be re-reviewed by one or more of the original referees, and acceptance of your manuscript will depend on your addressing satisfactorily the reviewers' major concerns. Please also note that Development will normally permit only one round of major revision.

We are aware that you may be experiencing disruption to the normal running of your lab that make experimental revisions challenging. If it would be helpful, we encourage you to contact us to discuss your revision in greater detail. Please send us a point-by-point response indicating where you are able to address concerns raised (either experimentally or by changes to the text) and where you will not be able to do so within the normal timeframe of a revision. We will then provide further guidance. Please also note that we are happy to extend revision timeframes as necessary.

Please attend to all of the reviewers' comments and ensure that you clearly highlight all changes made in the revised manuscript. Please avoid using 'Tracked changes' in Word files as these are lost in PDF conversion. I should be grateful if you would also provide a point-by-point response detailing

© 2022. Published by The Company of Biologists under the terms of the Creative Commons Attribution License (https://creativecommons.org/licenses/by/4.0/).

how you have dealt with the points raised by the reviewers in the 'Response to Reviewers' box. If you do not agree with any of their criticisms or suggestions please explain clearly why this is so.

Reviewer 1

Advance summary and potential significance to field

The manuscript describes gene expression patterns in the shoot apical meristem (SAM) of Populus trees by single nuclei RNA sequencing. This is the first time this type of analysis is performed in Populus, providing insight into functioning of the apical meristem in the rather complex context of a forest tree. Gene expression is validated by other methods for two genes (APL and LAX2), and functionally for one gene (LAX2). The data is clearly presented and the manuscript is well written.

Comments for the author

I have a few concerns and questions that are listed below.
1. 18 cell clusters were identified on the basis of the sequencing of the single nuclei. It was a bit surprising that the identity of four cell clusters remained unknown. Is this possibly due to the rather small number of marker genes that were used for the identification of the cell clusters? Use of a higher number of marker genes or comments on this issue would be informative.
2. An important part of the manuscript is based on comparison of the scRNA sequencing data between the current data in Populus SAM and published scRNAseq data on corresponding tissues in Arabidopsis. While the current manuscript analysed one Populus replicate sample, resulting in identification of 8,324 high-quality nuclei, the Arabidopsis paper (Zhang et al. 2021, Developmental Cell) reported on two replicates from the SAM as well as comparison to a leaf sample, resulting in identification of 36,643 high-quality cells of the shoot apices and leaves . I have several questions on this comparative transcriptomic analysis.
2A. There are several sources of error in the comparative gene expression analysis. There are large differences in the sequencing depth as well as the type of RNA used for the two studies (nuclear RNA in the Populus dataset and whole cell RNA in the Arabidopsis dataset). Furthermore, identification of the orthologous genes is never straightforward especially when it is done automatically for a large number of genes. What are the implications of these issues for the data interpretation?
2B . Why is the clustering of the data in Figure 3A so different from what was published by Zhang et al. (2021) in Developmental Cell ? For Arabidopsis, the data should be the same in both analyses.
2C. It is difficult to understand the statement on lines 197-198 " Only two clusters enriched in stress responding genes and one small group of cells were specific to Arabidopsis (Fig. S3 A, B). These clusters were removed for further analysis". According to figure S3A, three clustered (4,8,9) were removed as they hould not be present in Populus. But these clusters can be seen in figure 3 even though they are much smaller in Populus than in arabidopsis. Furthermore would not it be more interesting to focus on the differences, and hence on the clusters that differ between Arabidopsis and Populus ?
2D. The comparative analysis resulted in identification of putative new, "less characterized conserved genes implicated in phloem differentiation" such as HB33, NAC057, NAC075 and LHL1. On the basis of Arabidopsis gene expression databases, ANAC075 is also expressed in xylem, and LHL1 seems to have quite ubiquitous expression pattern. This does not of course exclude an important role in phloem differentiation, but some kind of validation of these results would be informative. Similarly, some kind of validation would have been very interesting to see for the transcription factors AUXIN RESPONSE FACTOR 2, CYTOKININ-RESPONSIVE GROWTH REGULATOR, HOMEOBOX PROTEIN 16 and NAC089 that were identified as unique to vascular development in Populus SAM. These are members of large gene families, and correct identification of the orthologs might be challenging.
2E. For an unfamiliar reader, it is not clear (lines 253-255) how one can see in Figs. S7 and S8 "genes whose gene expression changes significantly in phloem and xylem only in Populus (Tables S11 and S12; Figs. S7 and S8)" . Tables S11 and S12 are not clear either with first a list of Populus genes without annotations followed by a longer list of Arabidopsis genes with annotations.
2F. It is not clear in the "integrated" data where the procambial cells reside.

© 2022. Published by The Company of Biologists under the terms of the Creative Commons Attribution License (https://creativecommons.org/licenses/by/4.0/).

It says on lines 277-280 that “The PC cluster identified in Populus likely contains the procambial dividing cells ongoing their differentiation to phloem and xylem. However, in tune with the trajectory followed in Arabidopsis (Zhang et al., 2021), clusters 0 and 8 contain the procambial cells that are the precursors for the phloem and xylem formation.” If I understand this correctly the clusters 0 and 8 might not represent the diving, first specified procambial cells. I think this issue needs more clarification. The authors themselves mention that the procambium remains one of the most understudied plant tissues.
But have they managed to identify the early procambial cells and not only the phloem and xylem precursor cells? The expression of genes in Figure 4B have highest expression in the cluster 8 but which is not restricted to the cells in this cluster. It is of course possible that purely procambium specific genes don’t exist, or that the first, diving procambial cells are so few that it is not possible to identify them. It would have been anyway interesting to know how these genes in the “integrated” clusters 8 and 0 behave in the original clustering. Why not analyse more in detail gene expression in the VC-specific sub-cluster from the combined PC and VC clusters 4, 6, 10, and 16 that is shown to the right in figures 2C and D?
Minor issues Lines 228-234: For the sake of reproducibility, it is good to share scripts or code that they used for binning for trajectory analysis on github. And may be with the set seed option defined.
Line 275. Unclear where the information for the procambial marker genes (that are not involved in cell division) can be found.
Lines 308-309. In root, Monopteros is expressed in the protoxylem elements. The same holds true for Phabulosa and CNA/AtHB15.
Line 346. What is meant by “increased xylem length” ?
Fig 4B-D: missing the colour code Figure 6A. which lax line is used for this data? Or is the data combined for the two lines?
Line 314. Remove number "4" after the reference Ochando et al., 2008
-Better resolution is desired in the gus images.
-The genotyping of the LAX2 crispr line #1 seems inadequate as the two alleles for LAX2 are identical, which is highly unlikely. Line #1 is either heterozygous for mutation in LAX2 or the other mutated LAX2 allele has not been successfully identified.

<u>Reviewer 2</u>

Advance summary and potential significance to field

The paper submitted by Conde et al uses single cell sequencing to assess developmental trajectories in the apices of poplar. It goes on to compare data generated here with a previous study in which Arabidopsis shoot apex development was assessed using a similar method. While these parts of the manuscript are interesting, the final section, which aims to validate candidates, in my view is not of sufficient quality in its current form to see this manuscript published in development.

Comments for the author

Minor:
-There are some egregious oversights in terms of referencing. This is by no means an extensive list, but there are missing/incorrect citations for BP, STM
(line 126), PXY (line 135), ANT (line 137), KAN and FIL (line 144), PDF, HDG FDH (line 160), HB8 (line 162)…… I could go on. Citing Shi (2021), and Zhang (2021), for gene expression patterns published alongside a large edifice of molecular genetics spanning the last 25 years falls well short of the required standard.
-Lines 248-252: Can a cluster containing MP and PXY rally be considered genes for treachery element differentiation? PXY represses xylem differentiation. The role of MP is less clear, but at least one paper has described MP as an activator of PXY. The expression maxima of these genes is generally considered to be the xylem side of the cambium.

Major:
-Validation of the single cell sequencing is somewhat problematic. The authors make bold statements about the function of some of the analysed genes, e.g.

© 2022. Published by The Company of Biologists under the terms of the Creative Commons Attribution License (https://creativecommons.org/licenses/by/4.0/).

assigning LHL1 to phloem differentiation (Line 247). However these examples are never followed up with verification. Instead, the authors focus on APL and LAX2.
APL is well known as a regulator of phloem identity, and here the authors use the hairy root system to show that in poplar hairy roots, APL is expressed in the phloem. This cannot act as validation for transcriptome experiments in the shoot apex. The authors need to characterise the expression of multiple genes.
Poplar transformation is time consuming, so I suggest RNA in situ hybridization in the shoot apex would be appropriate. It would better support the experimental programme if some genes that apparently differ between Arabidopsis and poplar were included among those tested.
The section on LAX2 is stronger, but a better characterisation of expression at the shoot apex would strengthen the author's arguments. The authors have only looked at two lines, and this is not really enough to draw conclusions particularly given the levels of variation in plantlets derived from tissue culture. A minimum of 5 lines should be analysed, and ideally each with a greater n than 4.

Reviewer 3

Advance summary and potential significance to field

The authors applied the high throughput single nuclei RNA sequencing technique to poplar shoot apical meristem. In addition to single-cell transcriptome atlas of the poplar shoot apical meristem, the authors provided a pseudotime trajectory analysis of various differentiating tissues. They also compared the single-cell RNA seq data with corresponding data in Arabidopsis (i.e. comparison between woody perennial poplar and annual herbaceous Arabidopsis thaliana (Zhang et al., 2021)). Finally, the authors carried out functional studies with a few selected genes found from the analysis: they characterized the expression pattern of LAX2 and APL in poplar, and by generating via gene editing a double KO with two loci of LAX2 they showed a role for LAX2 in poplar xylem differentiation. This article contains wealth of gene expression information in poplar SAM, and as such is a good resource for scientists using poplar as a model.

Comments for the author

I don't have any major concerns, but several minor requests and suggestions:
1. The authors used a filtration function in Seurat that is setting the thresholds manually. Doesn't this cause bias? Since there are many other cell filtration methods, how did the authors confirm that this filtration is justified for further analysis?
2. The Arabidopsis SAM sequencing data is from protoplasts while the poplar SAM sequencing data is from nuclei, did the authors take this into consideration during data integration and find differentially expressed genes when comparing developmental trajectories within these two species?
3. From comparison of these two set of single-cell RNA seq data (Arabidopsis and poplar), especially from trajectory analysis, except for listing the differentially expressed genes, did the author found some general differences in tissue differentiation processes of these two species?
4. In material and methods part, is it possible to show exactly which part of the shoot apex did the authors took for the single nuclei sequencing (i.e. showing with an image)?
5. line 95-97, the author described they filtered bad quality cells with a minimum 1000 UMI count and a maximum of 7000 UMI count. In figure S1 the author described they filtered the cells with a minimum nFeature of 1000 and a maximum nFeature of 7000, did they use the same threshold for both nUMI and nFeatures?
The authors should explain clearer.
6. Did the cell cycle related genes affect the clustering?
7. Figure 1c, it would be more illustrative to draw borders rather than adding pots to show the location of different tissues in Populus apex.
8. Figure 2A and 3A, since there are too many Umaps with different cell groups, it is better to label the identity of each cluster, similar to the labels in Figure 1A. Figure 2A, what is the identity of subcluster3?
9. Figure 2A and 2C, it is a bit confusing, if the clusters in Figure 2C are the same as in figure 2A. It would be more reader-friendly to draw lines to show which groups of cells within the proliferating cells are extracted and combined with MC, EC or VC respectively, for reclustering and trajectory analysis.

© 2022. Published by The Company of Biologists under the terms of the Creative Commons Attribution License (https://creativecommons.org/licenses/by/4.0/).

10. In supplementary figure S4, S5, S7, and S8, it is better to add trajectory directions in the heatmap.
11. line 328-329, the GUS expression of LAX2 promoter looks interesting but from the cross section, the resolution is not high enough to address that LAX2 expresses in “the very first layers of the secondary xylem”
12. Line 135: correct reference for PXY expression is Fisher & Turner Curr Biol 2007
13. Line 137: correct reference for AINTEGUMENTA expression is Randall et al Biology Open 2015
14. Since the paper contains analysis of phloem transcriptome, it might be good to compare it to the very recently published high-resolution single cell phloem transcriptome (Roszak et al, Helariutta lab).

First revision

<u>Author response to reviewers' comments</u>

Reviewer: 1

Comments to the Author

The manuscript describes gene expression patterns in the shoot apical meristem (SAM) of Populus trees by single nuclei RNA sequencing. This is the first time this type of analysis is performed in Populus, providing insight into functioning of the apical meristem in the rather complex context of a forest tree. Gene expression is validated by other methods for two genes (APL and LAX2), and functionally for one gene (LAX2). The data is clearly presented and the manuscript is well written. I have a few concerns and questions that are listed below.

Major concerns:

1. 18 cell clusters were identified on the basis of the sequencing of the single nuclei. It was a bit surprising that the identity of four cell clusters remained unknown. Is this possibly due to the rather small number of marker genes that were used for the identification of the cell clusters? Use of a higher number of marker genes or comments on this issue would be informative.

    For this revised manuscript, we further explored the annotation of uncharacterized clusters by evaluating additional tissue-specific markers identified in single-cell transcriptomics studies published since our first submission (**e.g.*****, DOI: 10.1093/plphys/kiab489). We also further assessed the functional annotation of*** de novo ***gene markers identified for the uncharacterized cluster 0 (Table S3), which contains a large number of cells. More specifically, cells of this cluster contain transcriptome signatures of stem cells, such as an enrichment in the expression of KNAT2 and LSH3 (Figure 1, DOI: 10.1016/j.devcel.2021.02.021). Moreover, the expression of RPL is restricted to cells in cluster 0. This gene regulates the rib zone's cell division and growth (DOI: 10.1016/j.devcel.2016.08.013). HB13 is also a marker of cluster 0. In Populus stem, it has been described as expressed in undifferentiated parenchyma tissue (DOI: 10.1007/s00299-002-0476-6). Based on this new evidence, we annotated cluster 0 as ground -*** i.e., ***the tissue located below the stem cells in the apex, which also includes the rib meristem. These cells are highly represented in the apex. Also, the vascular cambium arises when the cells of the interfascicular parenchyma of the ground tissue located between the vascular bundles of the apex dedifferentiate and divide, connecting the procambium and completing the radial arrangement of the vascular cambium.***

    More careful examination of published single-cell transcriptome data also allowed us to refine the annotation of other clusters. For instance, the use of protoplasts in the single-cell analysis of developing leaves failed to identify the cluster corresponding with trichomes, as these cells are not isolated in the process. Thus, we did not include the analysis of very well-characterized regulators of trichome development in our first analysis, such as GLABROUS/GLABRA1 (GL1), GL2, and TRIPTYCHON (https://doi.org/10.1093/plphys/kiab489; https://doi.org/10.1016/j.devcel.2021.02.021). Trichomes are highly represented in the tissue used in our study, the poplar apex (Figure

© 2022. Published by The Company of Biologists under the terms of the Creative Commons Attribution License (https://creativecommons.org/licenses/by/4.0/).

S11). The expression of these genes is restricted to cluster 3 (Figure 1-R1). Accordingly, we reannotated this cluster as "trichomes".

The careful exploration of the remaining unannotated clusters 7, 9, and 14, did not point to a clear and definitive cell type. The presence of unannotated clusters is a common observation in single-cell transcriptome studies, as they may not include a sufficient number of cells to support identifying a specific cell type. These clusters may also include novel or rare cell types that have not yet been characterized in a woody perennial plant like Populus. Nonetheless, data presented in the manuscript (Figure 1) suggests that they may be transitioning cells related to proliferating, epidermal, and meristem cells.

We added the following sentences to the results and discussion sections and modified Figure 1 accordingly:

"In Arabidopsis, the use of protoplasts in the single-cell analysis of developing leaves failed to identify the cluster corresponding with trichomes, as these cells are not isolated in the process (https://doi.org/103/plphys/kiab489; https://doi.org/10.1016/j.devcel.2021.02.021). The expression of well characterized regulators of trichome formation, such as GLABROUS/GLABRA1 (GL1), GL2, and TRIPTYCHON (TRY) (10.1038/nrm1404) was restricted to cluster 3 and, accordingly, we annotated it as TRI (Fig. 1A-D). Finally, we also assessed the functional annotation of de novo identified marker genes identified for the cluster 0 (Table S3), which contains a large number of cells. More specifically, cells of this cluster contain transcriptome signatures of stem cells, such as an enrichment in the expression of KNOTTED-LIKE FROM ARABIDOPSIS THALIANA 2 (KNAT2) or LIGHT SENSITIVE HYPOCOTYLS 3 (LSH3) (Fig. 1B) (https://doi.org/10.1016/j.devcel.2021.02.021). Moreover, the expression of RPL is restricted to a group of cells located in cluster 0 (Fig. 1B). This gene regulates the rib zone's cell division and growth (DOI: 10.1016/j.devcel.2016.08.013). HB13 is also a marker of cluster 0. In Populus stem, it has been described as expressed in undifferentiated parenchyma tissue (DOI: 10.1007/s00299-002-0476-6). Based on this, we annotated cluster 0 as GMC - i.e., the tissue located below the stem cells in the apex, which also includes the rib meristem. These cells are highly represented in the apex. Also, the vascular cambium arises when the cells of the interfascicular parenchyma of the ground tissue located between the vascular bundles of the apex dedifferentiate and divide, connecting the procambium and completing the radial arrangement of the vascular cambium."

"Our results also suggest that nuclei isolation overcomes the limitation of exploring the specific transcriptomic of trichomes when using microfluidic approaches. The plant material used in the present study contains an abundant representation of these cells (Fig. S11)."

"The presence of unannotated clusters is a common observation in single-cell transcriptome studies, as they may not include a sufficient number of cells to support identifying a specific cell type. In our study, the careful exploration of the unannotated clusters 7, 9, and 14, did not point to a clear and definitive cell type. These clusters may also include novel or rare cell types that have not yet been characterized in a woody perennial plant like Populus. Nonetheless, the dot-plot of the well-characterized genes (Fig. 1B) suggests that they may be transitioning cells related to proliferating, epidermal, and meristem cells."

2. An important part of the manuscript is based on comparison of the scRNA sequencing data between the current data in Populus SAM and published scRNAseq data on corresponding tissues in Arabidopsis. While the current manuscript analysed one Populus replicate sample, resulting in identification of 8,324 high-quality nuclei, the Arabidopsis paper (Zhang et al. 2021, Developmental Cell) reported on two replicates from the SAM as well as comparison to a leaf sample, resulting in identification of 36,643 high-quality cells of the shoot apices and leaves. I have several questions on this comparative transcriptomic analysis.

   2A. There are several sources of error in the comparative gene expression analysis. There are large differences in the sequencing depth as well as the type of RNA used for the two studies (nuclear RNA in the Populus dataset and whole cell RNA in the Arabidopsis dataset). Furthermore, identification of the orthologous genes is never straightforward especially when it

© 2022. Published by The Company of Biologists under the terms of the Creative Commons Attribution License (https://creativecommons.org/licenses/by/4.0/).

is done automatically for a large number of genes. What are the implications of these issues for the data interpretation?

Indeed, the application of single-cell transcriptomics is relatively recent in plants. Even more uncommon is the use of nuclei as an alternative to overcome the limitations of isolating protoplasts from difficult-to-dissociate materials such as the Populus SAM. Here we used nuclei due to the difficulties of dissociating cells and isolating protoplasts from the inner layers of the SAM. While extending the tissue dissociation/protoplast isolation procedures could have resulted in an increase in cells released, adverse effects on cell viability and potential changes in transcriptional activity such as the introduction of stress-induced transcriptional artifacts have been found to occur (DOI: 10.1016/j.molp.2020.10.012). For woody species, this issue is only magnified by the presence of thick cell walls and compounds resistant to degradational stressors. The use of nuclei not only circumvents these issues but allows for the optimization of their use in microfluid devices. Naturally, concern has been raised about whether snRNA-sq adequately captures accurate and representative transcriptomic information. Several articles comparing datasets derived from both methods concluded that, while generally a higher median number of genes per cell and higher total expressed genes are found when utilizing protoplasts, there is a very high correlation between datasets generated by both methods. For instance, snRNA-seq reflects the observed root transcriptome generated by whole-tissue and protoplast single-cell analysis (https://doi.org/10.1016/j.molp.2021.01.001). Furthermore, a growing body of literature indicates that snRNA-seq achieves comparable results to scRNA-seq (DOI: 10.1681/ASN.2018090912, DOI: 10.1371/journal.pone.0209648), and the feasibility of integrating datasets from cells and nuclei (DOI: 10.1038/s41587-020-0469-4).

We agree that well-established gene orthology may be challenging to determine, especially in species with a complex evolutionary history of whole-genome duplications (as is the case of Populus) or limited genome resources and annotation. The generation of a one-to-one gene list from the two species is a key step before data integration. As the reviewer points out, it highly impacts the outcome of further downstream analyses such as trajectory inference. Despite this difficulty, we strongly believe in the power of single-cell data integration to compare developmental programs between species. In the present work, we applied an extensive and rigorous gene orthology analysis using protein sequence data from 93 species. This analysis allowed us to generate a high-confidence one-to-one gene list containing 9,827 Arabidopsis and Populus genes. Identifying shared expression programs among species, such as the one shown in our research work, is very useful in refining gene orthology. New methods that consider complex orthology relationships are emerging to integrate single-cell databases from different species, which aims to solve these limitations (DOI: 10.1101/2021.09.25.461790).

While we used the expression of 8,324 high-quality nuclei to identify the cell types in the SAM, we admit that this power may decrease when focusing on cell types that are not highly represented in the sample, such as phloem cells. In the future, we will complement this study with samples collected under different conditions and timepoints to evaluate the role of the circadian clock in vascular development, as highlighted recently (DOI: 10.1016/j.celrep.2022.111059).

We added the following sentence to the discussion section:

"Naturally, concern has been raised about whether snRNA-sq adequately captures accurate and representative transcriptomic information. Several articles comparing datasets derived from both methods concluded that, while generally a higher median number of genes per cell and higher total expressed genes are found when utilizing protoplasts, there is a very high correlation between datasets generated by both methods. For instance, snRNA-seq reflects the observed root transcriptome generated by whole-tissue and protoplast single-cell analysis (https://doi.org/10.1016/j.molp.2021.01.001). Furthermore, a growing body of literature indicates that snRNA-seq achieves comparable results to scRNA-seq (DOI: 10.1681/ASN.2018090912, DOI: 10.1371/journal.pone.0209648), and the feasibility of integrating datasets from cells and nuclei (DOI: 10.1038/s41587-020-0469-4).".

© 2022. Published by The Company of Biologists under the terms of the Creative Commons Attribution License (https://creativecommons.org/licenses/by/4.0/).

2B. Why is the clustering of the data in Figure 3A so different from what was published by Zhang et al. (2021) in Developmental Cell? For Arabidopsis, the data should be the same in both analyses.

Figure 3A shows the clusters obtained after clustering the integrated dataset derived from Populus and the vasculature subset of the Arabidopsis dataset published by Zhang et al., 2021 (as shown in Figures 4C and 6A of their publication) after applying Seurat's integration pipeline.

For the integration, we first recovered the raw data from the clustered data provided by Zhang et al. 2021. The raw data does not include any information regarding cluster (or cell-type) identity or the distribution of cells in the UMAP space. Next, we integrated both datasets using their raw data (count matrices), followed by clustering, resulting in the image shown in Figure 3A. Therefore, the UMAP plot in 3A shows clusters obtained for the integrated dataset. We split the plot by species to show the similar distribution of cells in the UMAP space and the proportion of cells of each sample in each cluster.

A comparison of the Populus dataset relative to the clustered Arabidopsis data (Zhang et al. 2021) was made and is shown in Figure S2. In the figure, we used the vasculature subset of the Arabidopsis data from Zhang et al. (2021). The UMAP plot is identical to the one shown by Zhang et al. (2021) in Figures 4C and 6A, except for differences in the colors used to highlight each cluster.

2C. It is difficult to understand the statement on lines 197-198" Only two clusters enriched in stress responding genes and one small group of cells were specific to Arabidopsis (Fig. S3 A, B). These clusters were removed for further analysis". According to figure S3A, three clustered (4,8,9) were removed as they should not be present in Populus. But these clusters can be seen in figure 3 even though they are much smaller in Populus than in arabidopsis. Furthermore, would not it be more interesting to focus on the differences, and hence on the clusters that differ between Arabidopsis and Populus?

We apologize for the confusing way this is described in the manuscript. After the integration and after applying the filtering parameters shown in figure S3, we excluded two clusters. These two clusters were only present in Arabidopsis. Gene Ontology analysis showed that these two clusters were enriched for stress-responsive genes. It is not possible to infer if these two clusters are only present in Arabidopsis due to differences in the plant material or if they occur because of the induction of expression of stress-responsive genes due to the protoplasting process. But their enrichment for stress-responsive genes led us to take a conservative approach and exclude them from further analysis, rather than making conclusions about what may be an experimental artifact. The final cluster that excludes them is shown in Figure S3 and Figure 3A. After generating a new clustering visualization, they are renumbered. Thus, the numbers of clusters removed in previous steps can be seen in the final UMAP visualization.

In this study, we focused on identifying common mechanisms of the vascular development between Arabidopsis and Populus and on identifying putative pathways specific to Populus. Although it may be interesting, we did not explore the specific clusters identified in Arabidopsis.

We added the following sentence to the results section in regard to this comment:

"Three clusters specific to Arabidopsis were excluded during the integration step, two of which were enriched for stress-responding genes, and a small cluster (Fig. S3 A, B). It is not possible to infer whether the clusters containing stress-responsive genes are only present in Arabidopsis due to differences between the species, or if they are the result of the induction of expression of stress-responsive genes due to the protoplasting process."

2D. The comparative analysis resulted in identification of putative new, "less characterized conserved genes implicated in phloem differentiation" such as HB33, NAC057, NAC075 and LHL1. On the basis of Arabidopsis gene expression databases, ANAC075 is also expressed in xylem, and LHL1 seems to have quite ubiquitous expression pattern. This does not of course exclude an important role in phloem differentiation, but some kind of validation of these results would be informative. Similarly, some kind of validation would have been very interesting to see for the transcription factors AUXIN RESPONSE FACTOR 2, CYTOKININ-RESPONSIVE GROWTH REGULATOR, HOMEOBOX PROTEIN 16 and NAC089 that were identified as unique to vascular development in Populus SAM. These are members of large gene families, and correct identification of the orthologs might be challenging.

© 2022. Published by The Company of Biologists under the terms of the Creative Commons Attribution License (https://creativecommons.org/licenses/by/4.0/).

We agree that the genes specifically associated with vascular development in Populus are of particular interest. In the future, functional gene characterization will be performed to elucidate the role of these genes in primary and secondary vascular development. We are already generating transgenic lines with perturbation in the expression of some of these genes. Their functional characterization and the understanding of the molecular mechanisms behind their regulation will be part of future research.

2E. For an unfamiliar reader, it is not clear (lines 253-255) how one can see in Figs. S7 and S8 "genes whose gene expression changes significantly in phloem and xylem only in Populus (Tables S11 and S12; Figs. S7 and S8)". Tables S11 and 12 are not clear either with first a list of Populus genes without annotations followed by a longer list of Arabidopsis genes with annotations.

In the sentence, we refer to the genes that showed expression associated with the trajectory toward xylem and phloem differentiation, only in Populus.

We agree that the gene nomenclature of Populus and Arabidopsis homologs is confusing. For this study, we generated a table of Populus-Arabidopsis homologs created by combining the high-confidence one-to-one ortholog gene list from our phylogenomic analysis and the Populus annotation information available in Phytozome. This table (Table S6) contains 23,732 Populus genes with the corresponding homolog in Arabidopsis. After the integration, the pipeline uses the gene IDs in the column "Arabidopsis" to explore the expression of Arabidopsis and Populus genes. To differentiate the expression of Populus paralogs that share a common Arabidopsis homolog, a dot followed by a number was added to the Arabidopsis ID of the corresponding Arabidopsis homolog (Table S6). For this reason, in the supplementary tables generated after the integration, in the "Populus" column, an Arabidopsis ID, followed by a dot and a number can be seen when the Populus gene has a homolog in Arabidopsis. The real Populus ID can be inferred by searching that gene ID in Table S6. Only when the Populus gene does not have an Arabidopsis homolog associated in Table S6, the Populus ID appears in the column "Populus". For that reason, in Tables S11 and S12, and Figures S7 and S8, Populus genes are named in some cases with the Arabidopsis IDs followed by a dot and a number, or with the Populus IDs in others. This is now described in the results section as described below:

"To create a one-to-one homolog genes list required for the integration, we applied a phylogenomic approach to defining a high-confident gene list of 9,842 Arabidopsis and Populus pairs of homologs (Table S5). The expression of these genes was used to integrate the single-cell expression data of Populus and Arabidopsis shoot apex vasculature. Homology between the remaining genes of Populus (not present in the one-to-one homolog genes list) and Arabidopsis's genes was established based on the most recently inferred relationships, available in Phytozome (Goodstein et al., 2012) (Table S6). After the data integration, we used this complete list to explore conserved and divergent pathways. This table (Table S6) contains 23,732 Populus genes with the corresponding homolog in Arabidopsis. After the integration, the pipeline uses the gene IDs in the column "Arabidopsis" to explore the expression of Arabidopsis and Populus genes. To differentiate the expression of Populus paralogs that share a common Arabidopsis homolog, a dot followed by a number was added to the Arabidopsis ID of the corresponding Arabidopsis homolog (Table S6). For this reason, in the supplementary tables generated after the integration (Table S7 to Table S15), in the "Populus" column, an Arabidopsis ID, followed by a dot and a number can be seen when the Populus gene has a homolog in Arabidopsis. The actual Populus ID can be inferred by searching that gene ID in Table S6. Only when the Populus gene does not have an Arabidopsis homolog associated in Table S6, the Populus ID appears in the column "Populus".

2F. It is not clear in the "integrated" data where the procambial cells reside. It says on lines 277-280 that "The PC cluster identified in Populus likely contains the procambial dividing cells ongoing their differentiation to phloem and xylem. However, in tune with the trajectory followed in Arabidopsis (Zhang et al., 2021), clusters 0 and 8 contain the procambial cells that are the precursors for the phloem and xylem formation." If I understand this correctly, the clusters 0 and 8 might not represent the diving, first specified procambial cells. I think this issue needs more clarification. The authors themselves mention that the procambium remains one of the most understudied plant tissues. But have they managed to identify the early

© 2022. Published by The Company of Biologists under the terms of the Creative Commons Attribution License (https://creativecommons.org/licenses/by/4.0/).

procambial cells and not only the phloem and xylem precursor cells? The expression of genes in Figure 4B have highest expression in the cluster 8 but which is not restricted to the cells in this cluster. It is of course possible that purely procambium specific genes don't exist, or that the first, diving procambial cells are so few that it is not possible to identify them. It would have been anyway interesting to know how these genes in the "integrated" clusters 8 and 0 behave in the original clustering. Why not analyse more in detail gene expression in the VC-specific sub-cluster from the combined PC and VC clusters 4, 6, 10, and 16 that is shown to the right in figures 2C and D?

In the integrated data of the vascular cells, we identified three clusters corresponding to the proliferating cells (PC). Dividing cells have a specific transcriptome signature. However, in addition to containing the proliferating cells of the procambium that are undergoing differentiation to phloem and xylem, these clusters may potentially also contain other dividing cells of the vasculature such as xylem or phloem cells undergoing further stages of differentiation. Hence, we can't determine that PC of vasculature are procambial cells exclusively. However, as suggested by the trajectory of the vascular cells (Figure S3C), and the trajectories observed in Arabidopsis (DOI: 10.1016/j.devcel.2021.02.021), clusters 0 and 8 contain the procambial cells that are the precursors for the phloem and xylem formation. At the time of sampling, they were not undergoing cell division, and hence, are characterized by the high expression of markers of procambial cells, as the ones described in the manuscript. As the reviewer mentioned, the expression of these genes is significantly induced in clusters 0 and 8, but their expression is not restricted to these cells. In figure 4, the expression of some of these genes is shown in the overall clustering shown in Figure 1A, obtained from the whole population of cells of the apex. The clusters mentioned by the reviewer shown in Figure 2C and D are of great interest and represent the Populus cells used to integrate the data with Arabidopsis in our study. In this research work, we explored the potential of the interspecific single-cell data integration because we believe in its potential to explore differences and similarities in cell differentiation programs between species that will be very useful to unravel questions about the acquisition of evolutionary innovations of high interest in agriculture and forestry.

We added the following sentence to the results section to clarify this point:

"In the integrated data of the vascular cells, we identified three clusters corresponding to the proliferating cells (PC) (Fig. 3A, B). Dividing cells have a specific transcriptome signature, and hence, they are clustered together during the data analysis. In addition to containing the proliferating cells of the procambium that are undergoing differentiation to phloem and xylem, these clusters may contain other dividing cells of the vasculature such as xylem or phloem cells undergoing further stages of differentiation. Hence, we can't determine that PC of vasculature are procambial cells exclusively. However, as suggested by the trajectory of the vascular cells (Fig. S3C) and the trajectories detected in Arabidopsis (https://doi.org/10.1016/j.devcel.2021.02.021), clusters 0 and 8 are transcriptionally similar to proliferating cells, and they contain the precursors for the phloem and xylem formation. However, these cells were not dividing at the time of sampling, based on the expression of cell-cycle marker genes (Fig. 3B)."

Minor concerns:

1. Lines 228-234: For the sake of reproducibility, it is good to share scripts or code that they used for binning for trajectory analysis on github. And may be with the set seed option defined.

   We agree with the reviewer that this is essential for the reproducibility of the study. The code necessary to reproduce the results described is now deposited in GitHub at https://github.com/KirstLab/scRNA-seq_vasculature_poplar_apex. A seed value was set in all scripts before the generation of the results presented in the manuscript and is described in all scripts deposited -n GitHub.

   Note that a large fraction of the results shown in the manuscript were obtained using Asc-Seurat (v.2.1), which is an interactive web interface. Therefore, generating a source code for this part of the analysis is impossible, and reproducibility requires repeating the execution manually. Nonetheless, the parameters to reproduce the analysis are shown in Figure S1 of the manuscript, and Asc-Seurat is publicly available. The integration of the two datasets was also performed in Asc-Seurat, using default parameters.

   We made available the Populus clustered dataset obtained in our analysis that can be loaded in Asc-Seurat. Since this is a large file, we deposited it separately in FigShare (DOI:

© 2022. Published by The Company of Biologists under the terms of the Creative Commons Attribution License (https://creativecommons.org/licenses/by/4.0/).

10.6084/m9.figshare.20321787). The link to access the rds files is included in the manuscript. While the files are under embargo until the final acceptance of the manuscript, reviewers can access the content by using the following private link: https://figshare.com/s/90f2df9685bb4a3eb958. The Arabidopsis dataset was provided to us by Zhang et al., 2021. To reproduce the results, readers would need to contact the authors of that study to acquire the clustered dataset.

2. Line 275. Unclear where the information for the procambial marker genes (that are not involved in cell division) can be found.
   We hope that we have clarified this confusing part of the manuscript by adding the paragraph indicated in point 2F.

3. Lines 308-309. In root, Monopteros is expressed in the protoxylem elements. The same holds true for Phabulosa and CNA/AtHB15.
   Thank you very much for pointing this out. These observations support the expression patterns observed in our study for those genes in Populus.

4. Line 346. What is meant by “increased xylem length”?
   We have clarified this in the manuscript. According to the paper cited (10.1093/aob/mcx091), xylem length is the length of a straight line traced from the last procambium cell layer to the inner xylem cells facing the center of the stem in cross-sections.

5. Fig 4B-D: missing the colour code
   We fixed this issue in the new version of figure 4.

6. Figure 6A. which lax line is used for this data? Or is the data combined for the two lines?
   We apologize for not being clear about this. We performed a complete phenotypical assay using three CRISPR/Cas9 mutants, and the results from each line are shown in the new version of figure 6.

7. Line 314. Remove number “4” after the reference Ochando et al., 2008
   We fixed this in the new version of the manuscript.

8. Better resolution is desired in the gus images.
   We included high-resolution images in the new version of figure 4. We believe this may be an issue when converting the final pdf file in the initial submission.

9. The genotyping of the LAX2 crispr line #1 seems inadequate as the two alleles for LAX2 are identical, which is highly unlikely. Line #1 is either heterozygous for mutation in LAX2 or the other mutated LAX2 allele has not been successfully identified.
   In the present study, we use the hybrid Populus tremula × alba INRA clone 717 1B4. The two alleles of each gene can be tracked by observing the mismatches between the alleles from Populus alba and tremula. However, the valuable resource that we have used for this purpose (https://www.aspendb.org) is being partially rebuilt, and this information will not be available for reviewers or the readers, and we don’t know when it will be. For this reason, we excluded this line and used two other additional lines with a clear biallelic mutation for both copies of the LAX2 gene.

Reviewer 2:

Comments to the Author

The paper submitted by Conde et al uses single cell sequencing to assess developmental trajectories in the apices of poplar. It goes on to compare data generated here with a previous study in which Arabidopsis shoot apex development was assessed using a similar method. While these parts of the manuscript are interesting, the final section, which aims to validate candidates, in my view is not of sufficient quality in its current form to see this manuscript published in development.

© 2022. Published by The Company of Biologists under the terms of the Creative Commons Attribution License (https://creativecommons.org/licenses/by/4.0/).

Major concerns:

1. Validation of the single cell sequencing is somewhat problematic. The authors make bold statements about the function of some of the analysed genes, e.g. assigning LHL1 to phloem differentiation (Line 247). However these examples are never followed up with verification. Instead, the authors focus on APL and LAX2. APL is well known as a regulator of phloem identity, and here the authors use the hairy root system to show that in poplar hairy roots, APL is expressed in the phloem. This cannot act as validation for transcriptome experiments in the shoot apex. The authors need to characterise the expression of multiple genes. Poplar transformation is time consuming, so I suggest RNA in situ hybridization in the shoot apex would be appropriate. It would better support the experimental programme if some genes that apparently differ between Arabidopsis and poplar were included among those tested.

   The application of RNA in situ hybridization in Populus is challenging due to the complex history of whole-genome duplications, chromosomal rearrangements, and tandem duplications that occurred in the genome, a resulted in a large number of paralogous genes (DOI: 10.1126/science.1128691). Paralogous genes can often develop a divergent function and expression pattern due to relaxed selective pressure on one or more copies of the duplicated gene (DOI: 10.1093/molbev/msu050).

   Despite the fact that we used several well-known markers to support the annotation of cell populations found in the Populus apex, after the initial reviews, we performed RNA in situ ***hybridization in an attempt to further validate the clustering. Procedure were performed following this protocol (http://plantlab.caltech.edu/html/protocols.html), which has been successfully used to characterize the expression of genes in Arabidopsis SAM (DOI:*** 10.1073/pnas.0900843106***). Probes were designed for six genes, all of which were*** de novo ***gene markers for clusters identified in our analysis (Table S3). Due to the high similarity to paralogs, probes were designed to target the less conserved regions in their transcripts' 5' or 3' UTR regions. Unfortunately, we generally detected a strong and unspecific background signal in our samples, given the results obtained from the antisense and sense probes. We believe in the strength of this technique, but extensive optimization will be necessary to generate optimal results that allow for robust conclusions. Despite these difficulties, we could confidently validate that the expression of the gene Potri.014G152000, identified as a marker of the epidermis (Table S3), is more highly expressed in the epidermis at the Populus shoot apex organs (new version of Figure 5).***

   As the reviewer mentioned, Populus transformation is time-consuming, usually requiring more than one year to generate enough plant material for proper characterization. For this reason, we used the hairy root transformation system based on the concept that gene markers of a specific tissue present in both the aerial part and the root can be validated more rapidly with this system. It should be noted that roots have been used extensively as model tissue to identify the molecular mechanism of primary and secondary vascular system development (e.g., DOI: 10.1038/s41586-018-0837-0).

   Overall, we believe that the combination of well-known markers and the validations we performed strongly supports the annotation of the Populus shoot apex cell populations identified in the present study. This data is a valuable resource to identify regulators of cell differentiation, as demonstrated by the functional characterization performed in this study.

   We have updated figure 5, results and materials, and methods according to this new data generated.

2. The section on LAX2 is stronger, but a better characterisation of expression at the shoot apex would strengthen the author's arguments. The authors have only looked at two lines, and this is not really enough to draw conclusions, particularly given the levels of variation in plantlets derived from tissue culture. A minimum of 5 lines should be analysed, and ideally each with a greater n than 4.

   A total of six lines were genotyped to identify mutations in the targeted regions of the genome. Four lines were selected for exploring changes in development, but only three were used in the latest and more complete phenotypical experiment due to the issues described previously (Reviewer 1, #9). In this experiment, five plants were characterized for each genotype. Because of the labor-intensive and time-consuming challenges of generating transgenic lines in Populus, 2-3 independent Cas9 mutant lines have been typically described in publications that characterize the function of genes in Populus, in

© 2022. Published by The Company of Biologists under the terms of the Creative Commons Attribution License (https://creativecommons.org/licenses/by/4.0/).

high-impact journals (e.g., DOI: 10.1016/j.cub.2022.05.023, DOI: 10.1073/pnas.1912434117, DOI: 10.1016/j.cub.2019.06.003).

We have updated figure 6, results and materials, and methods sections according to this new data generated.

Minor concerns:

1. There are some egregious oversights in terms of referencing. This is by no means an extensive list, but there are missing/incorrect citations for BP, STM (line 126), PXY (line 135), ANT (line 137), KAN and FIL (line 144), PDF, HDG, FDH (line 160), HB8 (line 162)…… I could go on. Citing Shi (2021), and Zhang (2021), for gene expression patterns published alongside a large edifice of molecular genetics spanning the last 25 years falls well short of the required standard.

   We apologize for this issue. We updated the list of references in the new version of the manuscript.

2. Lines 248-252: Can a cluster containing MP and PXY rally be considered genes for treachery element differentiation? PXY represses xylem differentiation. The role of MP is less clear, but at least one paper has described MP as an activator of PXY. The expression maxima of these genes is generally considered to be the xylem side of the cambium.

   We agree that the expression of these genes is not specific to tracheary elements. We clarified in the text that these genes are associated with the trajectory of tracheary elements in our analysis but are regulators of xylem differentiation.

Reviewer 3:

The authors applied the high throughput single nuclei RNA sequencing technique to poplar shoot apical meristem. In addition to single-cell transcriptome atlas of the poplar shoot apical meristem, the authors provided a pseudotime trajectory analysis of various differentiating tissues. They also compared the single-cell RNA seq data with corresponding data in Arabidopsis (i.e. comparison between woody perennial poplar and annual herbaceous Arabidopsis thaliana (Zhang et al., 2021)). Finally, the authors carried out functional studies with a few selected genes found from the analysis: they characterized the expression pattern of LAX2 and APL in poplar, and by generating via gene editing a double KO with two loci of LAX2 they showed a role for LAX2 in poplar xylem differentiation. This article contains wealth of gene expression information in poplar SAM, and as such is a good resource for scientists using poplar as a model.

I don't have any major concerns, but several minor requests and suggestions.

Minor concerns:

1. The authors used a filtration function in Seurat that is setting the thresholds manually. Doesn't this cause bias? Since there are many other cell filtration methods, how did the authors confirm that this filtration is justified for further analysis?

   We deployed a filtering approach broadly used for single-cell data analysis employing Seurat. The main goal is to remove nuclei that (1) show a smaller number of expressed genes than the observed for the majority of the cell population, likely reflecting empty GEMs whose gene counts represent contamination by environment (leaked) RNA, or (2) nuclei with a higher number of expressed genes than the remaining cell population, probably composed by GEMs where more than one nuclei were captured. While the parameter values are arbitrarily defined, we chose these limits after careful evaluation of the distribution of nuclei according to the number of expressed genes (nFeature parameter) in a Violin plot generated in Asc-Seurat.

   We also tested different values for these parameters and observed the most significant impact when modifying the lower threshold of the distribution. More specifically, when reducing that threshold to values smaller than 1000, we detected new clusters composed predominantly of nuclei that are not enriched for markers described in the literature or that lack enrichment of new markers. All combined, these observations support the hypothesis that the excluded "nuclei" represent empty GEMs and/or GEMs containing primarily ambient RNA, which should be removed from the analysis.

2. The Arabidopsis SAM sequencing data is from protoplasts while the poplar SAM sequencing data is from nuclei, did the authors take this into consideration during data integration and find

© 2022. Published by The Company of Biologists under the terms of the Creative Commons Attribution License (https://creativecommons.org/licenses/by/4.0/).

differentially expressed genes when comparing developmental trajectories within these two species?

Microfluid-based single cell and single nuclei transcriptomics are very recent techniques in plants and so far, the number of comparisons between both approaches is limited. As explained in point 2A of the responses to reviewer 1, existing data shows that there is a high correlation between the data obtained using both methods. However, using nuclei overcomes some of the significant limitations of using protoplasts to isolate individual cells, particularly from tissues such as the Populus apex. It is worth mentioning that when using nuclei, the transcriptome needs to be mapped against unmatured transcripts of the species of interest, keeping the introns as the unmatured transcript is highly present in the nuclei. When taking this under consideration, data obtained from both approaches have shown a high correlation (DOI: 10.1038/s41587-020-0469-4).

3. From comparison of these two set of single-cell RNA seq data (Arabidopsis and poplar), especially from trajectory analysis, except for listing the differentially expressed genes, did the author found some general differences in tissue differentiation processes of these two species?
Overall, the integration of the vascular cells data showed high conservation of the processes between both species, with most of the genes associated with the vascular tissue differentiation in Populus also present in Arabidopsis. Moreover, the general expression patterns along the trajectories of the regulators in Populus and Arabidopsis are similar (Figure S4 and S5), suggesting that not only many genes but also their expression is conserved during vascular differentiation.

4. In material and methods part, is it possible to show exactly which part of the shoot apex did the authors took for the single nuclei sequencing (i.e. showing with an image)?
We created a supplementary figure (Fig. S11) showing the specific portion of the Populus apex used in the present study.

5. line 95-97, the author described they filtered bad quality cells with a minimum 1000 UMI count and a maximum of 7000 UMI count. In figure S1 the author described they filtered the cells with a minimum nFeature of 1000 and a maximum nFeature of 7000, did they use the same threshold for both nUMI and nFeatures? The authors should explain clearer.
As the reviewer pointed out, the phrase explaining the filtering approach needed improvement. We incorrectly stated that UMI was used as the criteria for filtering. Instead, filtering was performed using nFeatures and not the UMI counts. Even though those two measures are strongly correlated, they represent different aspects of the data - UMIs represent the number of molecules detected in a cell, and nFeature the number of genes detected per cell. The necessary corrections were made to the original text.

6. Did the cell cycle related genes affect the clustering?
It is important to consider that when analyzing sing-cell or nuclei transcriptomic data, the pipeline uses the most variable genes to generate the cell clustering, grouping the cells based on the similar expression pattern of these most variable genes in the sample. Cell division results in a specific transcriptome signature. Unsurprisingly, in our data, as observed in Arabidopsis (DOI: 10.1016/j.devcel.2021.02.021), cells undergoing cell division are clustered together. These cells must be carefully characterized, as they contain all dividing cells of the sample, undergoing different cell type differentiation, as indicated by the expression of well-known markers. After this careful analysis, as shown in Arabidopsis and now in Populus, dividing cells can be reclustered together with the corresponding differentiated tissue, such as epidermis or vasculature.

7. Figure 1c, it would be more illustrative to draw borders rather than adding pots to show the location of different tissues in Populus apex.
To keep the clarity of the image in Figure 1C, we kept the dots to show the location of the different tissues. We made the changes suggested by the reviewer in figure S11, to indicate the position of each tissue.

8. Figure 2A and 3A, since there are too many Umaps with different cell groups, it is better to label the identity of each cluster, similar to the labels in Figure 1A. Figure 2A, what is the identity of subcluster3?

© 2022. Published by The Company of Biologists under the terms of the Creative Commons Attribution License (https://creativecommons.org/licenses/by/4.0/).

Figure 2A shows the sub-clustering of proliferating cells (clusters 5, 11 and 12 of Figure 1A). We don't know precisely the identity of subclusters 1 and 3 of this sub-clustering, but they don't seem to be epidermal, vascular, trichomes, or mesophyll dividing cells. They may correspond to the cell division of more undifferentiated meristem cells. Figure 3A shows the reclustering of the corresponding proliferating cells with mesophyll, epidermal or vascular cells. Apart from the proliferating cells, we don't exactly know the nature of these subgroups of cells inside each tissue. They may represent different stages of cell differentiation or, as it may be the case for epidermal cells, different location of the tissue in different organs (e.g., developing leaves or stem). In the case of the vasculature, the subclusters correspond to the dividing cells and the differentiated tissues such as phloem and xylem. We further explore them after the data integration.

9. Figure 2A and 2C, it is a bit confusing, if the clusters in Figure 2C are the same as in figure 2A. It would be more reader-friendly to draw lines to show which groups of cells within the proliferating cells are extracted and combined with MC, EC or VC respectively, for reclustering and trajectory analysis.
Yes, clusters of figure 2A show the subclustering of proliferating cells (clusters 5, 11 and 12 of Figure 1A). The expression patterns of well-known markers allow the identification of which subclusters of the proliferating cells correspond to MC, EC and VC. So, figure 2C shows the reclustering of MC, EC and VC identified in figure 1A, with their corresponding proliferating cells identified in figure 2A (MC-subcluster 0, EC-subcluster 2 and 6, VC-subcluster 4).

10. In supplementary figure S4, S5, S7, and S8, it is better to add trajectory directions in the heatmap.
We included the trajectory direction in the heatmaps of these figures.

11. line 328-329, the GUS expression of LAX2 promoter looks interesting but from the cross section, the resolution is not high enough to address that LAX2 expresses in "the very first layers of the secondary xylem"
We have included a new version of figure 5, that includes high-resolution images that we hope help to clarify the expression patterns of LAX2.

12. Line 135: correct reference for PXY expression is Fisher & Turner Curr Biol 2007
We corrected the reference in the new version of the manuscript.

13. Line 137: correct reference for AINTEGUMENTA expression is Randall et al Biology Open 2015.
We corrected the reference in the new version of the manuscript.

14. Since the paper contains analysis of phloem transcriptome, it might be good to compare it to the very recently published high-resolution single cell phloem transcriptome (Roszak et al, Helariutta lab).
We thank the reviewer for pointing our attention to this research work. We will perform this comparison in our follow-up work, when we will evaluate the plasticity of vascular development in Populus, and we have more power to establish comparison in such as a specific group of cells, during primary and secondary growth. We cited this and other similar works in the manuscript in the following sentence:
"The definition of cells in distinct layers and functional zones of the Populus shoot apex now offers researchers the opportunity to investigate, at unprecedented resolution, how stem cells differentiate into distinct cell types in the shoot of perennial species, and compare those mechanisms with the vast knowledge generated in the annual plants model species Arabidopsis, where the root phloem differentiation mechanisms have been finely dissected (10.1126/science.aba5531; 10.1038/s41477-022-01178-y)".

© 2022. Published by The Company of Biologists under the terms of the Creative Commons Attribution License (https://creativecommons.org/licenses/by/4.0/).

Second decision letter

MS ID#: DEVELOP/2022/200632

MS TITLE: Single-cell analysis of the shoot apex vascular system differentiation in Populus

AUTHORS: Daniel Conde, Paolo M. Triozzi, Wendell J. Pereira, Henry W. Schmidt, Kelly M. Balmant, Sara A. Knaack, Arturo Redondo-Lopez, Sushmita Roy, Christopher Dervinis, and Matias Kirst

I have now received all the referees reports on the above manuscript, and have reached a decision. The referees' comments are appended below, or you can access them online: please go to BenchPress and click on the 'Manuscripts with Decisions' queue in the Author Area.

The overall evaluation is positive and we would like to publish a revised manuscript in Development, provided that the referees' comments can be satisfactorily addressed. Please attend to all of the reviewers' comments in your revised manuscript and detail them in your point-by-point response. If you do not agree with any of their criticisms or suggestions explain clearly why this is so. If it would be helpful, you are welcome to contact us to discuss your revision in greater detail. Please send us a point-by-point response indicating your plans for addressing the referee's comments, and we will look over this and provide further guidance.

Reviewer 2

Advance summary and potential significance to field

Conde et al's revised manuscript is improved, however, major concerns raised in the previous round of reviews have not been addressed.

Comments for the author

Major issues:

1) Validation of APL using the hairy root system.

This is a fundamental flaw in that sequencing data from the shoot apex is validated in a root system. Shoot data should be validated in the shoot as the regulatory mechanisms as mechanisms governing the two tissues differ.

2) Validation of further factors.

Validation of Potri.014G152000 is a step forward in demonstrating the robustness of the method, but this still leaves only 2 expression patterns that support the sequencing data (Potri.014G152000, and LAX2). Regarding RNA in situ hybridization the authors state that it is challenging but that, "extensive optimization will be necessary to generate optimal results that allow for robust conclusions." To my mind this is a necessary step, the alternative being generation of further transgenic lines, or LCM plus qPCR (https://doi.org/10.1371/journal.pgen.1000320). The reason that this is important is illustrated elsewhere in the manuscript. For example, in line 249 the authors associate T5L1 with tracheary elements. In Arabidopsis, T5L1 has a role in regulation of proliferation in the vascular cylinder. It may be true that in poplar, T5L1 is associated with TE's, but in situ is therefore needed to confirm this. Generally, validation of expression patterns of multiple genes is required. To my mind, a minimum of 6 would is necessary to draw robust conclusions.

Reviewer 3

Advance summary and potential significance to field

The authors applied the high throughput single nuclei RNA sequencing technique to poplar shoot apical meristem. In addition to single-cell transcriptome atlas of the poplar shoot apical meristem, the authors provided a pseudotime trajectory analysis of various differentiating tissues. They also

© 2022. Published by The Company of Biologists under the terms of the Creative Commons Attribution License (https://creativecommons.org/licenses/by/4.0/).

compared the single-cell RNA seq data with corresponding data in Arabidopsis (i.e. comparison between woody perennial poplar and annual herbaceous Arabidopsis thaliana (Zhang et al., 2021)). Finally, the authors carried out functional studies with a few selected genes found from the analysis: they characterized the expression pattern of LAX2 and APL in poplar, and by generating via gene editing a double KO with two loci of LAX2 they showed a role for LAX2 in poplar xylem differentiation. This article contains wealth of gene expression information in poplar SAM, and as such is a good resource for scientists using poplar as a model. (Note: same text as in the first review report)

Comments for the author

The authors have addressed well my previous concerns. I have one suggestion though to change the title: Single-nuclei transcriptome analysis of the shoot apex vascular system differentiation in Populus.
There are many types of single cell or nuclei analyses, not just transcriptome.

Second revision

Author response to reviewers' comments

Reviewer: 2

Comments to the Author

Conde et al's revised manuscript is improved, however, major concerns raised in the previous round of reviews have not been addressed.

Major concerns:

1. Validation of APL using the hairy root system. This is a fundamental flaw in that sequencing data from the shoot apex is validated in a root system. Shoot data should be validated in the shoot as the regulatory mechanisms as mechanisms governing the two tissues differ.
   We used the hairy root system as an alternative for validating results in a woody species because (1) the generation of* Populus *transgenics is extremely time-consuming (particularly when compared to model plants like Arabidopsis), (2) because of the commonality between tissues in shoots and roots, and (3) because roots have now been used extensively to study the vascular cambium development, suggesting an overlap in their development programs. We used APL, a very well-characterized regulator of phloem formation (sieve elements and companion cells) in both shoot and root (https://doi.org/10.1038/nature02100). APL is a well established marker of these cell types in roots, stems, and apices (https://doi.org/10.1038/nature02100, https://doi.org/10.1038/nature02100, https://doi.org/10.1016/j.devcel.2021.02.021. To satisfy the reviewer's concern, we have excluded the hairy root validation of the expression of APL (Potri.010G174100) and included the citation of a recent article that showed it is a marker of sieve elements and companion cells in the phloem of Populus in the stem (https://doi.org/10.1186/s13059-021-02537-2).

2. Validation of further factors. Validation of Potri.014G152000 is a step forward in demonstrating the robustness of the method, but this still leaves only 2 expression patterns that support the sequencing data (Potri.014G152000, and LAX2). Regarding RNA in situ hybridization the authors state that it is challenging but that, "extensive optimization will be necessary to generate optimal results that allow for robust conclusions." To my mind this is a necessary step, the alternative being generation of further transgenic lines, or LCM plus qPCR (https://doi.org/10.1371/journal.pgen.1000320). The reason that this is important is illustrated elsewhere in the manuscript. For example, in line 249, the authors associate T5L1 with tracheary elements. In Arabidopsis, T5L1 has a role in regulation of proliferation in the vascular cylinder. It may be true that in poplar, T5L1 is associated with TE's, but in situ is

© 2022. Published by The Company of Biologists under the terms of the Creative Commons Attribution License (https://creativecommons.org/licenses/by/4.0/).

therefore needed to confirm this. Generally, validation of expression patterns of multiple genes is required. To my mind, a minimum of 6 would is necessary to draw robust conclusions.

We are generating transgenic lines for functional analysis of previously uncharacterized genes in Populus development, including more GUS staining lines. However, due to the timeline required for their characterization, they will be part of future reports. We believe that the expression pattern of a good number of well-known marker genes led us to a robust annotation of the cell types identified in the present study. In the past year, a couple of research studies have been focused on applying the same technology to decipher cell-type specific transcriptomes in Populus stem or secondary xylem, using protoplasts (https://doi.org/10.1186/s13059-021-02537-2; https://doi.org/10.1111/jipb.13159). Chen et al. (2021) established a web server (https://scu-populus.shinyapps.io/scRNAPal/) to facilitate the use of their scRNA-seq data. Even considering that Populus stem and apex differ in their cell populations, both tissue types should have conserved expression patterns in several cell types such as epidermis, xylem, and phloem, even when the apex is enriched in primary vasculature while in the stem the secondary vascular system is predominant. To partially address concerns from the reviewer, we used their web server to explore the expression of marker genes for the epidermis, sieve elements, companion cells, and xylem identified in our study. We found that genes identified in our study to be present in phloem and xylem mother cells, sieve elements, companion cells, and epidermis, including APL, SEOR1, AIL5, or LAX2, are also markers for the same cell type in the stem (Table S2 of https://doi.org/10.1186/s13059-021-02537-2). We generated and included in the manuscript a new supplementary figure (Figure S12) with the expression data of these genes in both datasets, which we believe highlights the accuracy of our cell type annotation and the importance of the datasets generated for scientists using poplar as a model.

T5L1 was associated with the tracheary elements in our analysis, as well as in Arabidopsis using cells from shoot apex (https://doi.org/10.1016/j.devcel.2021.02.021). T5L1 has been associated with the regulation of protoxylem vessel formation in Arabidopsis (http://dx.doi.org/10.1016/j.cub.2015.10.051). A recent single-cell transcriptomic analysis in Populus secondary developing xylem showed that T5L1 was significantly induced in the cluster annotated as differentiating vessels, with respect to other clusters such as fibers or ray parenchyma (Supplemental Dataset 3 of http://dx.doi.org/10.1016/j.cub.2015.10.051). We agree that further functional characterization is required to finely define the role of T5L1 and other candidates identified in our work during vascular system formation in Populus.

Reviewer 3:

Comments to the Author

The authors applied the high throughput single nuclei RNA sequencing technique to poplar shoot apical meristem. In addition to single-cell transcriptome atlas of the poplar shoot apical meristem, the authors provided a pseudotime trajectory analysis of various differentiating tissues. They also compared the single-cell RNA seq data with corresponding data in Arabidopsis (i.e. comparison between woody perennial poplar and annual herbaceous Arabidopsis thaliana (Zhang et al., 2021)). Finally, the authors carried out functional studies with a few selected genes found from the analysis: they characterized the expression pattern of LAX2 and APL in poplar, and by generating via gene editing a double KO with two loci of LAX2 they showed a role for LAX2 in poplar xylem differentiation. This article contains wealth of gene expression information in poplar SAM, and as such is a good resource for scientists using poplar as a model. (Note: same text as in the first review report).

Minor concerns:

1. The authors have addressed well my previous concerns. I have one suggestion though to change the title: Single-nuclei transcriptome analysis of the shoot apex vascular system differentiation in Populus. There are many types of single cell or nuclei analyses, not just transcriptome.

© 2022. Published by The Company of Biologists under the terms of the Creative Commons Attribution License (https://creativecommons.org/licenses/by/4.0/).

We thank the reviewer for this suggestion. We believe that this new title defines more accurately what we did in this study, and we modified the title to: "Single-nuclei transcriptome analysis of the shoot apex vascular system differentiation in Populus."

Third decision letter

MS ID#: DEVELOP/2022/200632

MS TITLE: Single-cell analysis of the shoot apex vascular system differentiation in Populus

AUTHORS: Daniel Conde, Paolo M. Triozzi, Wendell J. Pereira, Henry W. Schmidt, Kelly M. Balmant, Sara A. Knaack, Arturo Redondo-Lopez, Sushmita Roy, Christopher Dervinis, and Matias Kirst
ARTICLE TYPE: Research Article

I am happy to tell you that your manuscript has been accepted for publication in Development, pending our standard ethics checks.

Reviewer 2

Advance summary and potential significance to field

For summary of advance, please see previous review.

Comments for the author

Removal of hairy root analysis improves the manuscript. Cross-referencing with existing datasets is helpful and provides confidence in the single cell data.

Reviewer 3

Advance summary and potential significance to field

This is very useful paper for labs studying vascular or SAM development in Poplar.

Comments for the author

I have no further suggestions.

© 2022. Published by The Company of Biologists under the terms of the Creative Commons Attribution License (https://creativecommons.org/licenses/by/4.0/).
